# Paternity Analyses for the Planning of SIT Projects against the Red Palm Weevil

**DOI:** 10.3390/insects14040326

**Published:** 2023-03-28

**Authors:** Silvia Belvedere, Silvia Arnone, Massimo Cristofaro, Alessandra La Marca, Alessio De Biase

**Affiliations:** 1 Department of Biology and Biotechnologies “Charles Darwin”, Sapienza University of Rome, Viale dell’Università 32, 00185 Rome, Italy; silvia.belvedere84@gmail.com; 2 ENEA C.R. Casaccia TERIN-BBC-BIC, Via Anguillarese 301, 00123 Rome, Italy; silvia.arnone@enea.it; 3 BBCA-Onlus, Via Angelo Signorelli 105, 00123 Rome, Italy; m.cristofaro55@gmail.com (M.C.); alm.ales27@gmail.com (A.L.M.)

**Keywords:** red palm weevil, microsatellite, paternity assignment, SIT

## Abstract

**Simple Summary:**

The red palm weevil *Rhynchophorus ferrugineus* is an invasive pest insect that feeds on more than 20 species of palms, many of which are widely grown for food and ornamental use. Due to its vast spread and the huge economic damage this pest can cause, it is very important to find sustainable and effective eradication strategies. Sterile insect techniques are biological control approaches based on the release of mass-reared sterilized males that could improve the likelihood of successful pest control. However, for the successful implementation of these approaches, it is necessary to develop a deep understanding of the insect’s mating system. For this purpose, we built a paternity assignment strategy based on previously developed microsatellite loci to help the future study of the reproductive mechanisms of this species in laboratory-controlled mating experiments. We evaluated the reliability of our genetic tool using a simulation approach that aimed to explore its resolution power in paternity tests, both in the laboratory and in nature.

**Abstract:**

The red palm weevil *Rhynchophorus ferrugineus* is an invasive pest from southeastern Asia and Melanesia that has spread widely across the Middle East and the Mediterranean Basin over the last 30 years. Its endophagous larvae cause huge amounts of damage to several palm tree species from the Arecaceae family. Many of these palms are economically important for agricultural and ornamental purposes. Therefore, a lot of attention has recently been focused on studying this species with the aim of identifying sustainable and effective eradication strategies. Sterile insect techniques are biological control strategies that are currently being investigated for their potential to eradicate this pest in selected invasion areas. Mating system features (e.g., polyandry and related features) can affect the success and suitability of these approaches. The main goal of this research was to assess the performance of a previously developed microsatellite panel in terms of the paternity assignment of progeny from laboratory mating experiments. Using a simulation approach, we evaluated the reliability of the microsatellite markers in the paternity tests both in complex laboratory experiment scenarios and on the progeny of wild-caught gravid females to help future studies on the RPW mating system. As a case study of the simulation results, we performed two double-mating experiments, genotyped the progeny and estimated the P_2_ values to compare to the expected progeny genotypes according to the crossing scheme of each experiment. The results of our simulations on laboratory experiments showed that it was possible to carry out paternity assignments for all progeny with reliable statistical confidence using our 13 microsatellites set. On the contrary the low genetic variability measured in red palm weevil populations in invaded areas made the resolution power of our loci too low to carry out paternity analyses on natural populations. Results of laboratory crossing were completely congruent with the expectations from the Mendelian laws.

## 1. Introduction

The red palm weevil *Rhynchophorus ferrugineus* (Olivier, 1790; Coleoptera, Dryophthoridae, hereafter referred to as RPW) is a polyphagous invasive pest insect that feeds on more than 20 species of palms in the Arecaceae family, many of which are widely grown for food and ornamental use (i.e., the Canary Island palm *Phoenix canariensis* Hort. ex Chabaud, the date palm *Phoenix dactylifera* L., the coconut palm *Cocos nucifera* L. and the oil palm *Elaeis guineensis* Jacq. [1,2]). Larval instars burrow into the trunks and/or basal leaf shoots of host plants and their perforations can reach the main meristem, causing leaves to fall and often resulting in the death of the palm tree [3,4,5].

The primary distribution of the weevil is southeastern Asia and Melanesia; however, since the 1980s, the RPW has spread across the Middle East and the Mediterranean Basin through the trade of infested palm trees [6,7,8]. It has also expanded its distribution range eastwards to China and Japan and westwards to the Caribbean [9,10,11,12].

As a result of its attacks on several palm species in the invaded countries, the spread of the RPW has caused huge economic damage. The RPW is registered in the A2 EPPO (European and Mediterranean Plant Protection Organization) as a quarantine pest [13] and is considered to be among the main pests of the date palm *P. dactylifera*, which is widely cultivated in the arid regions of the Middle East and North Africa as an important food resource and the main agricultural crop in desert habitats [14]. This palm species is also exported to many countries in the Mediterranean basin, where it is extensively used as an ornamental plant in urban centers and along coastal roads.

One of the key features of the widespread and harmful invasion of the RPW is the high reproductive success of the species, which has been broadly investigated in previous works on various reproductive parameters, such as oviposition period, the number of eggs laid and their hatching rate, survival at immature stages, the number of generations per year, the lifetime fecundity of adult females, etc. [10,15,16,17,18]. It should be pointed out that these parameters are strongly dependent on feeding substrates and environmental conditions (i.e., laboratory or field experiments, temperature, humidity, seasonality, geographic region, etc.) and also that they have shown huge variations between different studies. However, some studies have clearly indicated that females can oviposit hundreds of eggs at a time during long oviposition periods that last for several weeks and that the species could complete more than one generation per year [5,10,19]. On the other hand, many aspects of RPW reproductive biology have not yet been fully explained, so more in-depth studies on its mating system are needed in order to ground future management actions. For example, even though multiple mating in RPW has been observed in nature and more frequently in the laboratory [20], very little is known about its sperm storage and usage mechanisms. The occurrence of re-mating and the possibility of females retaining sperm from different mates (that is potentially available for insemination) could constitute critical factors influencing the success of management programs that are dependent on female mating, such as sterile insect techniques (SITs) [21]. SITs are biological control strategies based on the release of large numbers of mass-reared sterilized males that mate with wild females, thus inducing sterility and reducing the targeted population size. In addition, SITs can be applied to support classic biological control approaches to manage RPW: in fact, non-viable eggs laid by females that mated with irradiated males could be suitable substrates for the oviposition of egg parasitoids [22]. However, polyandry can affect the long-term stability of populations by playing a role in maintaining genetic variability and increasing the effective population size. These reflections are of particular interest for outbreak events of invasive species, such as the RPW [23]. For instance, within the context of eradication programs for damaging species, in areas where temporary residual populations could establish or cleared areas that could experience re-infestations, the occurrence of re-mating could strengthen the reproductive potential of the re-invading propagules in terms of their effective population size.

The occurrence of re-mating within a species does not necessarily translate into multiple paternity among the offspring of single females because of the existence of post-copulatory sexual selection mechanisms, which basically work at two levels [24,25,26]: female cryptic choice and sperm competition. The female selection of ejaculates is difficult to detect because it takes place within the female reproductive system. Sperm competition can occur both by preventing the female from mating again with others (via mating plugs, guarding, prolonged copulation, the induction of a refractory period in the female, etc.) and by obtaining sperm priority. Male adaptations for sperm priority comprise many behavioral and physiological components (such as sperm removal, stratification, last-in-first-out mechanisms, sperm dilution, the chemical or behavioral stimulation of the female, the evolution of particular sperm traits, etc.) [24] reported in the order Odonata [27] and in the mealworm beetle *Tenebrio molitor* L. [28], grasshopper *Locusta migratoria* L. and the tree cricket *Truljalia hibinonis* (Matsumura) [29,30], flies *Drosophila melanogaster* Meigen [31] and *Dryomyza anilis* Fallén [32]. As a consequence, estimating the degree of polyandry in a species involves considering the possibility of such post-mating sexual selection mechanisms [33,34,35,36] and the reproductive success advantages for the last male that mated with a female (the so-called last male sperm precedence), which is particularly common in insects.

Within this field, studies exploring the effects of mating order on fertilization success, which is classically analyzed as the proportional paternity of the second male (P_2_ value) [37], have often advanced our understanding of the relative influences of post-copulatory male–male competition and female choice on sexual selection, even within a laboratory context. This is of particular interest because evidence for the last male sperm precedence in RPW has come from laboratory double-mating experiments involving wild and sterilized males, which have found that females only produce viable progeny when the last male that mated with them was the wild-type [38,39]. Indeed, to evaluate the suitability of SIT strategies, a few experiments have been performed to test the effects of different γ ray doses on the reproductive physiology and mating behavior of the RPW. To determine the paternity of progeny from females that mated with multiple males, double-crossing experiments have been carried out by confining individual females with either wild-type males or γ-irradiated males. The results have shown that the progenies are almost exclusively produced by the sperm of the second male, suggesting that the last male sperm precedence commonly occurs in this species [39]. Therefore, despite the apparent complications in the reproductive behavior of females that have been observed in the field (i.e., polyandry and high levels of fertility), the results of laboratory experiments have shown that other features of the mating system of this species (i.e., the last male sperm precedence, high vitality and the ability of irradiated males to mate) could make the application of SITs possible.

On the other hand, when studying other aspects of reproductive biology, such as polyandry and the relative contributions of different males to broods, genetic parentage analysis is one of the most powerful and reliable approaches. In this case, the use of microsatellite markers (SSRs) as molecular tools to assess paternity within the context of laboratory-controlled mating experiments is the best way to test hypotheses about the mating systems of those species [40], such as the RPW, for which direct observations in the wild are difficult, particularly when cryptic sexual selection is suspected. Furthermore, the RPW generates large colonies of hundreds of individuals inside host palms. Nevertheless, the genetic structures of such colonies have not yet been studied, so it is impossible to attribute a priori the larvae found in hosts to their respective mothers or to know how many different broods are present. Consequently, it is hard to estimate how many candidate mothers and fathers should be considered, as well as the probability of finding them in the same palm or in the same neighborhood. Unfortunately, the number of potential parents and, even more, the proportion by which they are sampled are important variables in parentage analysis [40]. According to the above-mentioned reasons and to the knowledge at our disposal, conducting direct paternity analyses on natural RPW populations would be very challenging.

Therefore, the main goal of this research was to assess a previously developed SSR panel [41] in terms of achieving the paternity assignment of progeny obtained from laboratory mating experiments. We conducted power analysis simulations to evaluate the reliability of these microsatellite markers for paternity tests, both via complex laboratory experiments and on the progeny of wild-caught gravid females, in order to help future studies on the mating system of the RPW. In addition, we assessed whether the developed method could be used as an efficient tool when applied to populations in invaded areas, such as those in Italy. As a case study of the simulation results, we performed two double-mating experiments, genotyped the progeny and estimated the P_2_ values to compare to expected progeny genotypes according to the crossing scheme of each experiment.

## 2. Materials and Methods

### 2.1. Simulated Paternity Assignments

For the simulations of the paternity tests, we used a set of 13 previously developed microsatellite loci [41], which were selected for amplification success and polymorphism. Then, two additional loci (P4C2 and P1A3) were chosen from the literature [42]. In another study [43], we performed a preliminary analysis of genetic variability in natural RPW populations from primary and secondary distribution areas with the purpose of identifying well-differentiated source populations for the collection of individuals to be examined in laboratory experiments. It clearly emerged that there was a higher genetic variability among individuals from the primary distribution areas. Based on this background information, we focused our attention on Italian RPW populations with the aim of supporting future research on the mating system of this pest in invaded areas. Further, in view of the lower genetic variations that we detected compared to those in the primary distribution areas, we selected 23 individuals from a previously developed genotypic dataset [41], which were collected from several Italian regions. Using CERVUS v.3.0.6 software [44], we calculated the polymorphism information content (PIC) and exclusion probabilities of the loci in order to perform a power analysis of the microsatellite panel, which we used as a paternity analysis tool for Italian populations in which the maternal genotype was known. Using the same software but under the scenario of more complex laboratory mating experiments and possible studies on natural populations, two series of paternity analysis simulations were carried out.

Series 1: We conducted simulations of laboratory experiments in which females were given the opportunity to mate with several males according to the following parameters (Table 1):

- The number of candidate fathers (i.e., the number of males the females mated with) in each simulation was 2, 4, 6, 8 and 10;
- The number of offspring produced was 150 larvae (which represented a true estimate of the average successful reproductive rate of laboratory-reared females [16]);
- The proportion of candidate fathers sampled was 1.00 (which was assumed to genotype all males involved in the experiment);
- The proportion of typed loci was 0.99 (based on the almost total absence of missing data from previous experiments due to the previous optimization of conditions for DNA extraction and amplification);
- The proportion of genotyping errors was 0.01 (i.e., the standard estimated error rate in the production of genotypic microsatellite data [44]).

Series 2: We also conducted simulations of the paternity assignments of progeny from known wild mothers who mated in a natural environment (Table 2). This scenario referred to putative laboratory experiments involving adult samples of both sexes that were collected from Italian populations. Male individuals were treated as candidate fathers and females (which were assumed to have been fertilized just before capture) were reared until they had completed oviposition in order to analyze their progeny. In this experimental scenario, we not only tested the effects of higher numbers of candidate fathers (5, 10, 20, 30 and 40 sampled males) but also those of different proportions of candidate fathers sampled (0.25, 0.50, 0.75 and 1.00). Within the context of a natural population that satisfied the panmixia criterion, the potential fathers of the given progeny were theoretically all of the fertile males that were present. It should be noted that estimating the actual number of male individuals in a RPW population is rather complex as no population studies have been conducted to date in this regard. Furthermore, the spatial delimitation of populations can only be performed arbitrarily by considering the distribution of host palms within given areas and the dispersion capacity of the insect without being able to take into account the possible genetic structures of the populations (i.e., the presence of more or fewer isolated colonies on different palms). The simulation parameters relating to the quantity of offspring produced, the proportion of typed loci and the proportion of genotyping errors were kept the same as for series 1.

### 2.2. Laboratory Mating Experiments

#### 2.2.1. The Creation of Genetically Diverse Stocks of Individuals

Considering our dataset and taking into account data from the *coxI* mitochondrial marker, we found that most reciprocally genetically divergent populations were those from the Mediterranean Basin and Vietnam [41,43]. In order to maximize the probability of exposing females to males carrying different alleles, thus improving the resolution power of our system, we decided to create laboratory stocks of two distinct genealogical strains from individuals that were collected from the field using pheromone traps in Italy and Vietnam and then lab-reared using apple as a feeding substrate [45]. The Italian samples were collected in 2013 from central Italy (i.e., from the ENEA C.R. Casaccia, Rome, Lazio) and the Vietnamese samples were collected at the end of 2012 from Hanoi city (Hanoi Province) by collaborators. In the experiments, which were carried out in 2013, we only employed virgin males and second-generation reared females in order to avoid increasing homozygosity due to inbreeding. In order to ensure virginity, we isolated the cocoons before adult emergence.

#### 2.2.2. Mating and Rearing

**Figure 1:**
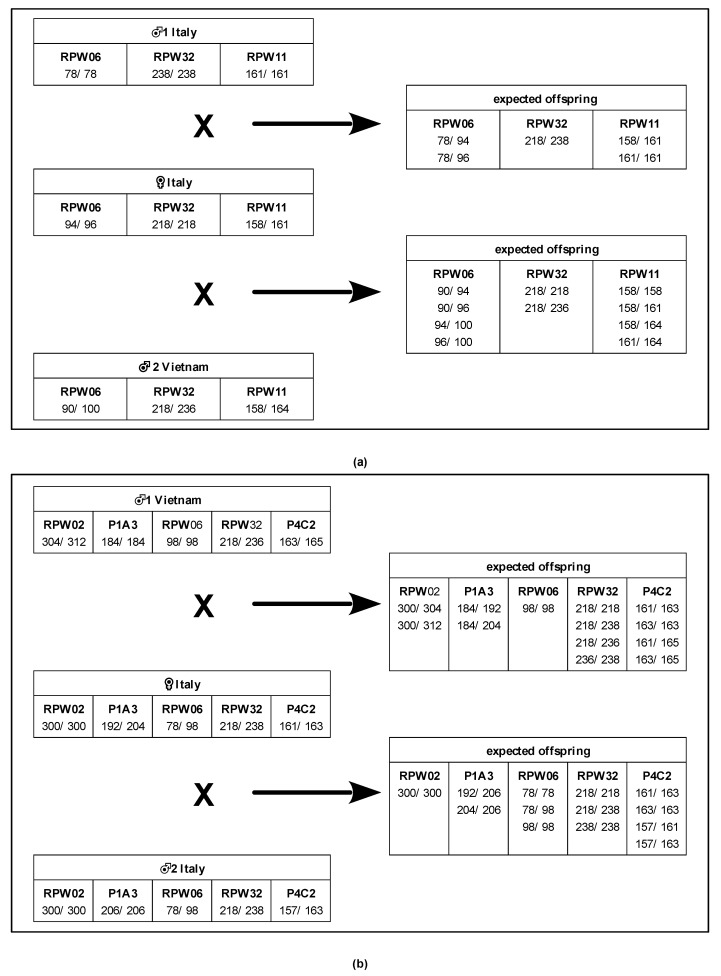
The genotypes of the mother and putative fathers and the expected genotypes for (**a**) experiment 1 and (**b**) experiment 2. The numbers refer to the allele dimensions.

We performed two mating experiments (Figure 1) in which one virgin female from the Italian line was confined in a plastic cage with a single male until copulation occurred. The first male was then removed and immediately replaced with a second before the female could start to oviposit any eggs. The second male was then kept in the cage with the female until a second copulation event took place. Since frequent pre-copulatory interactions do not necessarily lead to successful copulation events in this species [20], the mating events were assessed by careful visual observations based on the recording of the aedeagus in the female genital opening for at least one minute. In experiment 1, the first male introduced into the cage was from the Italian strain, while the second was from the Vietnamese line. In experiment 2, it was the opposite. This was carried out in order to evaluate any differences in paternal success due to possible female selection mechanisms driven by the genetic similarity between partners [46]. After mating, the males were immediately killed and stored in acetone [47] for subsequent genetic analysis, while the females were isolated in separate cages and provided with apple slices for food and oviposition substrates before they were also killed and stored in acetone. The laid eggs were checked daily and the larvae were then isolated to prevent cannibalism, which has been observed in previous experiments. Similarly, the hatching of each egg was recorded daily. The larvae were fed apple substrates and then killed when they reached dimensions of about 1 cm in order to allow for easy DNA extraction. After improving our DNA extraction protocol, we killed larvae of 1–2 mm to avoid cannibalism events.

#### 2.2.3. Genotyping and Paternity Analysis

Genomic DNA from the adults and offspring was isolated using a classical phenol/chloroform extraction protocol [48]. The microsatellite primer pairs and polymerase chain reaction (PCR) amplification parameters were described in detail in [41]. Appropriate controls were included in every amplification reaction to ascertain that neither the template nor primers were contaminated. The genotyping was based on the polymorphism of the fragment length of the fluorescently tagged DNA fragments using an ABI 3730XL sequencer with 400HD-ROX Size Standard (Applied Biosystems, Foster City, CA, USA) at the Macrogen Inc. facility in Korea. The microsatellite allele sizes were scored using MICROSATELIGHT [49] and visually checked using Peak Scanner^TM^ v.1.0 (Applied Biosystems) in order to identify possible genotyping errors.

We genotyped the mothers and candidate fathers using 15 microsatellite markers and analyzed them in order to identify the loci that were the most informative in terms of the paternity assignment in each experiment. We defined loci as “not diagnostic” when males 1 and 2 shared the same genotype and as “partially diagnostic” when the two males shared one allele and/or the female shared an allele with at least one of the males. Therefore, depending on which allele the mother and/or father transmitted to the progeny, the resulting filial genotype could be unambiguously assigned to one male. Finally, we defined loci as “totally diagnostic” when the two males did not share any alleles between each other or the female, so all expected progeny genotypes from each father were different. Following the genotyping protocol described earlier, we analyzed the progeny on all available totally diagnostic and some partially diagnostic loci to ensure good reliability in the paternity assignments, despite the possibility of genotyping errors.

The allelic size dataset was checked for numeric errors and null alleles at a 95% confidence interval using MICROCHECKER v.2.2.3 [50] and by comparing the expected genotypes of the progeny to the adult genotypes. Loci that showed evidence of the presence of null alleles were discarded from the subsequent analyses. For each experiment, the observed and expected heterozygosity, polymorphism information content (PIC), non-exclusion probabilities and combined non-exclusion probabilities (according to [51,52]) were calculated using CERVUS v.3.0.6 software [44]. The calculations were based on the observed allele frequencies of the 40 individuals from Italy and Vietnam (23 and 17 individuals, respectively) that had been genotyped for the preliminary genetic differentiation analysis. For this reason, we could not report any results for Hardy–Weinberg tests or null allele frequency estimation, given the artificial nature of the “source populations” of the samples involved. Parentage analyses were then performed for the two experiments using the same program, which calculated a likelihood ratio score for each candidate parent to identify the male that was most likely the true father of a particular offspring [44]. Critical likelihood values (LOD scores, i.e., the natural logarithm of the likelihood-odds ratio for each adult male) yielding 95% and 80% confidence in assignments were obtained via the simulations. We simulated 10,000 offspring, assuming an average mistyping error of 0.01 per locus. Paternity analyses were also conducted on the progeny of the two experiments using the Bayesian approach implemented in the PATRI software (PaTernity Inference [53]).

## 3. Results

### 3.1. Simulations

The CERVUS estimates of the allele number per locus, the number of typed individuals, PIC and non-exclusion probability for the second parent are shown in Table 3 based on the observed allele frequencies in the Italian sample. On average, the PIC values for most of the loci were below the 0.5 threshold for informative loci; therefore, only two loci (RPW06 and P4C2) were considered to be informative in Italian populations. However, the combined non-exclusion probability for the second parent was rather low (0.03), making our loci panel suitable for carrying out paternity analyses (for similar findings, also see [54,55]).

The results from the simulations in series 1 (i.e., the laboratory mating experiments with different numbers of males and a single virgin female) were promising (Table 4). The assignment rate was 100% at a 95% confidence level for paternity attribution when the mother was known and there were two, four or six candidate fathers, while it was 99% when there were eight candidate fathers. When there were 10 candidate fathers, the assignment rate decreased to 89% or below as the number of males increased.

The simulations in series 2 aimed to investigate the possibility of performing paternity assignments for the progeny of wild-caught gravid females and showed an overall low assignment rate at a 95% confidence interval (Table 5). As expected, the percentage of progeny attributed to relative fathers decreased with the proportion of candidate fathers sampled and when the effective male population size increased.

### 3.2. Laboratory MATING Experiments

#### 3.2.1. Mating and Rearing

During experiment 1, copulation started 6 min after the introduction of male 1 (Italian) and lasted for 2 min, while copulation with male 2 (Vietnamese) started only 1 min after it was introduced and also lasted for 2 min. In experiment 2, the first male (Vietnamese) mated with the female after 9 min and copulation lasted for 5 min, while male 2 (Italian) mated with the female after 4 min and copulation lasted for 1 min. For this reason, we decided to allow more mating events to occur in order to reach a comparable total amount of copulation time. Consequently, male 2 mated with the female two more times, spontaneously suspending copulation after 1 min both times and waiting 7 min between each copulation event.

The results of the female and male reproductive success are summarized in Table 6, while more detailed data concerning the two replicates are reported in the Supplementary Materials (Tables S1 and S2). In experiment 1, the female laid a total of 175 eggs in 29 days, during which we counted 14 distinct oviposition events. Out of the 127 hatched eggs, 81 were successfully reared until they achieved the target dimensions for DNA extraction and were then analyzed. In spite of the loss of many offspring, we were able to analyze at least two individuals from each oviposition event (Table S1), which represented a good sample of the progeny produced during the overall period of 29 days. To counteract the loss of progeny samples, we decided to put more effort into our DNA extraction protocol in order to efficiently extract DNA from very small larvae (1–2 mm), thus reducing the rearing time to just 2 or 3 days for experiment #2. During experiment 2, the oviposition period lasted 28 days and 17 oviposition events were recorded. From the total of 192 eggs that were laid, 132 hatched and only 7 larvae died (which were not suitable for the subsequent genetic analyses due to their very small size and decaying tissues). We were then able to analyze the remaining 125 larvae. Therefore, we obtained a very good sample of the viable progeny produced by the females. Additionally, the seven lost individuals originated from eggs with oviposition dates that were equally distributed throughout the overall oviposition period.

#### 3.2.2. Genotyping

Table 7 shows the genotypes of the parental generations from experiments 1 and 2, with the loci labelled according to their diagnostic power. After the selection of the most informative markers, the progenies were assayed at five microsatellite loci (RPW02, RPW06, RPW32, RPW11 and P1A3) for experiment 1 and six loci (RPW02, P1A3, RPW36, RPW06, RPW32 and P4C2) for experiment 2. We then excluded loci RPW02 and P1A3 from the paternity analysis of experiment 1 and RPW36 from the paternity analysis of experiment 2 because of the clear presence of null alleles (unpublished data).

Figure 1 shows the parental genotypes and expected genotypes for the offspring of the two candidate fathers for the selected loci in each experiment. We genotyped all available survived offspring, with a total of 81 specimens in experiment 1 and 125 specimens in experiment 2 (the genotypes are listed in the Supplementary Materials, Tables S3 and S4).

#### 3.2.3. Paternity Analysis

As shown in Table 8, the mean PIC value for experiments 1 and 2 was 0.562 and 0.672, respectively, which was above the 0.5 thresholds by which the loci were considered informative. The non-exclusion probability for the second parent (i.e., the probability of not excluding an individual that was not related to the tested progeny as a candidate parent) when the genotype of the other parent was known was 0.168 and 0.020 for experiments 1 and 2, respectively (Table 8).

These values were quite different and not significant because the non-exclusion probabilities were calculated based on the overall genotypic dataset of Italian and Vietnamese individuals that we characterized for the exploration of polymorphism levels, as previously stated (see Methods). As a consequence, these estimates were based on the allele frequencies of “artificial populations” consisting of individuals with different origins. It should be noted that for the paternity analyses, we instead selected the most informative loci on the basis of the particular genotypes of the adults we crossed; therefore, within the context of our experiments, the non-exclusion probabilities were lower than estimated.

The paternity analyses conducted using CERVUS to calculate the LOD scores (given by the natural logarithm of the overall likelihood ratio) revealed a clear pattern of sperm usage in terms of the last male that mated with a female. All of the 81 progeny produced by the female in experiment 1 were fathered by the second male (P_2_ value = 1.00), while in experiment 2, only 2 larvae were attributed to the first male compared to the 123 (98.4%) that were clearly fathered by the second male (P_2_ value = 0.98). For both experiments, we recorded an assignment rate of 100% at a 95% confidence interval and the LOD scores were always positive, ranging between 1.04 and 8.46 for experiment 1 and between 1.88 and 7.14 for experiment 2 (for details, see Tables S5 and S6 in the Supplementary Materials). As expected, the results from the PATRI software totally confirmed those from CERVUS, with a posterior probability of 1 for the assignment of each offspring to each father (for details, see Tables S7 and S8 in the Supplementary Materials). Interestingly, the two offspring of male 1 in experiment 2 came from eggs laid at distinct moments during the oviposition period (i.e., 5 and 18 days after the mating event).

## 4. Discussion

### 4.1. Simulations

In this study, we evaluated the resolution power of our microsatellite panel for future research by simulating different scenarios with an increasing number of potential fathers and a single mating female. The preliminary results of our simulations suggested that it was possible to carry out paternity assignments for all progeny with reliable statistical confidence using our 13 SSR set and crossing Italian RPW individuals, even when up to eight males were offered to the female. Given that a very small secondary contribution from the first male emerged in experiment 2, it could be important to only accept very small error rates in future paternity tests. In other words, the experimental designs of future studies must be considered important steps in order to assign the paternity of sperm with a high precision rate (nearly 100%), making this approach a good tool for cases of small contributions from more than one male. 

We also performed supplementary simulations that aimed to evaluate the paternity assignment rate with different proportions of sampled candidate fathers. This way, we could test the possibility of performing paternity assignments on the progeny of wild-caught pregnant females. As reasonably expected, the results from these simulations confirmed our suspicion that the low genetic variability measured in RPW populations in invaded areas, such as Italy, Greece and Spain [41,43], made the resolution power of our microsatellites too low to carry out paternity analyses on natural populations. The simulations also suggested that using this approach on natural Italian populations would produce a low rate of expected success in terms of paternity assignment, especially when the number of candidate fathers was high and the proportion by which they were sampled was less than 1. Indeed, under natural conditions, the dimensions of effective male populations could be very high (as males produce an attractant aggregation pheromone when colonizing a new host [56]), although it is very difficult (or unrealistic) to ensure the confidence in capturing the quasi-totality of males. However, it could be possible to achieve better and more reliable assignment rates in RPW populations with higher genetic variability (and more polymorphic markers), such as those in the primary distribution areas or from the Arab peninsula, which probably played a bridgehead role in the invasion dynamics of the RPW [43].

### 4.2. Paternity Analyses of Mating Experiments

As a case study for conducting paternity analysis using our SSR loci, we successfully performed two different double-mating experiments involving virgin RPW adults, which aimed to evaluate the relative contributions of two competing males in the progeny of a single female. The results showed that even though multiple mating is common in the sexual behavior of this species [57], the female tended to fertilize eggs with the sperm of only one male. This confirmed the evidence found by Musmeci et al. [39], which was derived using a different complementary approach to that adopted in this work. It is an interesting behavior and more specific experiments are needed to assess the RPW sperm usage mechanisms in order to discriminate the respective roles of sperm competition and cryptic female choice in the evolution of RPW reproductive behavior. Considering the effects of intra-specific variability, the results of the two experiments were very similar in terms of the oviposition duration period, the number of distinct oviposition events, the total number of eggs laid and the proportion of eggs hatched (Table 6). Moreover, even though we were able to analyze fewer larvae in experiment 1 than in experiment 2, larval mortality was equally distributed throughout the two oviposition periods and we could successfully sample the progeny of each oviposition event because of the higher post-natal mortality rate (survived larvae/hatched eggs = 36%). Consequently, we could conclude that the results of the two experiments were quite comparable.

Our pilot experiments were designed to maximize the genetic differences between individual parents and allow the genotypes of the offspring to be accurately calculated. From each crossing, we obtained more than 100 larvae that could be genotyped (apart from the few units mentioned above), which represented a large sample of individuals. The results of the genotyping were completely congruent with the expectations represented in Figure 1, which were derived from the Mendelian laws. Therefore, we are confident about the resolving power of the panel of microsatellite loci that was evaluated.

Additionally, the paternity analyses that were conducted on these experiments clearly indicated that our panel of SSR loci was adequate for performing this kind of analysis on offspring obtained in experimental settings with a success rate of nearly 100%. Therefore, the loci published in [41] could be useful markers for the design of laboratory experiments to study the mating system of this invasive species. Conversely, the levels of genetic variability observed in these markers among populations from invasion areas meant that we could not carry out paternity studies under natural conditions. However, this approach could be effective for studying the sperm competition mechanisms of other insect pest species that are targets of SIT strategies.

## 5. Conclusions

The use of microsatellite genotyping for parentage assignment is a common procedure in animals and our set of microsatellite markers proved to be a powerful tool for paternity assignment in laboratory experiments involving different males. The results of our simulation trial could help to deepen the study of the RPW mating system encouraging more complex experiments that consider more variables (e.g., timing, the duration and frequency of copulation in which females are exposed to many males, age and the physiological status of irradiated individuals), which could identify the mechanisms behind the post-copulatory last male precedence (female cryptic choice vs. sperm competition strategies) that has been suggested by previously cited studies.

## Figures and Tables

**Table 1:** The paternity analysis simulations in series 1.

Common Parameters	
Number of offspring/female	150
Proportion of sampled candidate fathers	1.00
Proportion of typed loci	0.99
Proportion of genotyping errors	0.01
Likelihood error rate	0.01 (default)
Minimum number of typed loci/individual	8.00
**Variable Parameters**	
Number of candidate fathers	2; 4; 6; 8; 10

**Table 2:** The paternity analysis simulations in series 2.

Common Parameters	
Number of offspring/female	150
Proportion of typed loci	0.99
Proportion of genotyping errors	0.01
Likelihood error rate	0.01 (default)
Minimum number of typed loci/individuals	8.00
**Variable Parameters**	
Number of candidate fathers	5; 10; 20; 30; 40
Proportion of sampled candidate fathers	0.25; 0.5; 0.75; 1.00

**Table 3:** The diagnostic power of the 13 polymorphic microsatellites in Italian populations.

Locus	N_A_^1^	N ^2^	PIC ^3^	NE-2P ^4^
RPW02	3	22	0.360	0.802
RPW03	4	22	0.405	0.758
RPW06	7	21	0.712	0.467
RPW11	2	22	0.208	0.896
RPW16	2	22	0.348	0.826
RPW20	2	22	0.370	0.815
RPW22	2	22	0.208	0.896
RPW25	2	22	0.318	0.841
RPW26	2	22	0.305	0.848
RPW32	2	22	0.330	0.835
RPW36	2	22	0.305	0.848
P4C2	4	22	0.617	0.593
P1A3	5	22	0.473	0.698

Note: ^1^ allele number; ^2^ the number of individuals; ^3^ polymorphism information content; ^4^ second parent non-exclusion probability (first parent known).

**Table 4:** The paternity assignment rate (%) of the series 1 simulations at a 95% confidence interval.

	Number of Candidate Fathers				
	2	4	6	8	10
**Assignment rate**	100%	100%	100%	99%	89%

**Table 5:** The paternity assignment rate (%) of the series 2 simulations, combining the different numbers of candidate fathers and the variable proportions of sampled candidate fathers at a 95% confidence interval.

		Number of Candidate Fathers				
		5	10	20	30	40
**Proportion of Sampled Candidate Fathers**	**0.25**	33%	19%	18%	15%	5%
	**0.50**	55%	33%	29%	24%	13%
	**0.75**	75%	59%	57%	49%	28%
	**1.00**	100%	89%	67%	49%	32%

**Table 6:** The reproductive parameters of experiments 1 and 2.

	Experiment 1	Experiment 2
**Oviposition Period (days)**	29	28
**Number of Oviposition Events**	14	17
**Number of Eggs Laid**	175	192
**Number of Eggs Hatched (%)**	127 (72.57%)	132 (68.75%)
**Number of Larvae that Survived**	81	125

**Table 7:** The criteria for the selection of the most informative loci in the paternity assignments in experiments 1 and 2.

	Experiment 1						Experiment 2					
Locus	Female		Male 1		Male 2		Female		Male 1		Male 2	
RPW02	300	300	300	300	292	292	300	300	304	312	300	300
RPW03	-	-	214	214	214	214	-	-	212	214	212	214
RPW06	94	96	78	78	90	100	78	98	98	98	78	98
RPW11	158	161	161	161	158	164	161	161	158	158	158	161
RPW13	-	-	172	172	172	172	-	-	172	172	172	172
RPW16	-	-	226	229	226	226	226	226	226	226	226	229
RPW17	-	-	222	222	219	222	222	222	219	222	222	222
RPW20	-	-	82	86	86	86	82	86	86	86	82	86
RPW22	-	-	159	163	159	159	163	163	159	163	163	163
RPW24	-	-	95	95	95	95	-	-	95	95	95	95
RPW25	-	-	99	99	99	99	-	-	99	99	99	99
RPW26	-	-	140	140	132	140	-	-	140	140	140	140
RPW32	218	218	238	238	218	236	218	238	218	236	218	238
RPW36	-	-	143	145	145	145	143	143	143	145	145	145
P1A3	184	204	192	204	184	184	192	204	184	184	206	206
P4C2	-	-	157	163	157	157	161	163	163	165	157	163

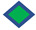
 Totally diagnostic loci: No shared alleles between the males or between the female and either male. 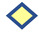 Partially diagnostic loci: Males had one shared allele and/or the female shared an allele with at least one of the males. 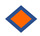 Not diagnostic loci: Males had the same genotypes. Not genotyped because the locus was excluded from the subsequent analyses on the basis of the male genotypes. The numbers refer to the allele dimensions.

**Table 8:** The parameters of the microsatellite loci that were analyzed in experiments 1 and 2.

Locus	k	N	H_o_	H_e_	PIC	NE-1P	NE-2P	NE-PP	NE-I	NE-SI
**Experiment 1**										
**RPW06**	13	38	0.553	0.850	0.823	0.472	0.307	0.130	0.042	0.341
**RPW11**	5	40	0.375	0.599	0.514	0.816	0.684	0.530	0.245	0.515
**RPW32**	3	40	0.325	0.391	0.349	0.925	0.803	0.676	0.414	0.660
**Mean/Combined**					0.562	0.357	0.168	0.047	0.004	0.116
						
**Experiment 2**										
**RPW02**	7	38	0.368	0.711	0.665	0.696	0.516	0.318	0.125	0.431
**P1A3**	13	40	0.450	0.816	0.789	0.525	0.351	0.157	0.055	0.361
**RPW06**	13	38	0.553	0.847	0.820	0.478	0.311	0.132	0.043	0.343
**RPW32**	3	40	0.350	0.406	0.360	0.919	0.796	0.668	0.400	0.649
**P4C2**	9	38	0.500	0.772	0.726	0.628	0.450	0.263	0.093	0.392
**Mean/Combined**					0.672	0.101	0.020	0.001	0.000	0.014

Note: k—allele number; N—the number of individuals; H_o_—observed heterozygosity; H_e_—expected heterozygosity; PIC—polymorphic information content; NE-1P—non-exclusion probability for first parent; NE-2P—non-exclusion probability for second parent; NE-PP—non-exclusion probability for parental pair; NE-I—non-exclusion probability for identity; NE-SI—non-exclusion probability for sib identity; Mean/Combined—the mean PIC value or the combined non-exclusion probability values.

## Data Availability

The data presented in this study are available in the Supplementary Materials (here).

## Supplementary Materials

The following are available online at https://www.mdpi.com/article/10.3390/insects14040326/s1, Table S1: The reproductive parameters of experiment 1, Table S2: The reproductive parameters of experiment 2, Table S3: The progeny genotypes from the three most informative loci of experiment 1 (ordered by oviposition event), Table S4: The progeny genotypes from the three most informative loci of experiment 2 (ordered by oviposition event), Table S5: The paternity assignment of experiment 1 using three microsatellite loci and Cervus v.3.0.6 software, Table S6: The paternity assignment of experiment 2 using three microsatellite loci and Cervus v.3.0.6 software, Table S7: The paternity assignment of experiment 1 using Patri software, Table S8: The paternity assignment of experiment 2 using Patri software.
