# Supplementary material for: Paternity Analyses for the Planning of SIT Projects against the Red Palm Weevil
Source: Insects. 2023 Mar 28;14(4):326. doi: 10.3390/insects14040326 (PMC10144754; doi:10.3390/insects14040326)
Supplement: Supplementary file 1 [file insects-14-00326-s001.zip › insects-2187801-supplementary.pdf]

Supplementary Material

Table S1. The reproductive parameters of experiment #1

Oviposition event	Oviposition date	# laid eggs	# hatched eggs	% hatching	# survived larvae
I	27.IX.13	10	7	70	3
II	30.IX.13	6	5	83	4
III	03.X.13	19	7	37	6
IV	04.X.13	8	7	88	4
V	07.X.13	29	28	97	21
VI	08.X.13	16	16	100	6
VII	10.X.13	6	4	67	3
VIII	11.X.13	8	4	50	4
IX	14.X.13	7	7	100	2
X	16.X.13	6	6	100	2
XI	18.X.13	12	11	92	9
XII	21.X.13	13	11	85	9
XIII	23.X.13	8	7	88	2
XIV	25.X.13	10	7	70	6

Table S2. The reproductive parameters of experiment #2

Oviposition event	Oviposition date	# laid eggs	# hatched eggs	% hatching	# survived larvae
I	17.I.14	13	8	61	8
II	20.I.14	40	31	77	30
III	21.I.14	9	9	100	9
IV	22.I.14	8	6	75	6
V	23.I.14	8	4	50	4
VI	24.I.14	1	1	100	1
VII	27.I.14	22	15	68	13
VIII	28.I.14	11	8	73	7
IX	30.I.14	14	3	21	3
X	31.I.14	4	3	75	2
XI	03.II.14	24	20	83	19
XII	04.II.14	7	5	71	4
XIII	05.II.14	6	2	33	2
XIV	10.II.14	13	10	77	10
XV	11.II.14	7	5	71	5
XVI	12.II.14	2	1	50	1
XVII	13.II.14	1	1	100	1

Table S3. The progeny genotypes from the three most informative loci of experiment #1 (ordered by oviposition event)

Event	Sample ID	RPW06		RPW32		RPW11	
I	IV3.L01	90	96	218	218	158	164
I	IV3.L02	90	94	218	218	161	164
I	IV3.L03	96	100	218	218	158	158
II	IV3.L04	94	100	218	236	158	158
II	IV3.L05	94	100	218	218	161	164
II	IV3.L06	90	94	218	218	158	158
II	IV3.L07	94	100	218	218	158	161
III	IV3.L08	90	96	218	236	158	161
III	IV3.L09	94	100	218	236	158	161
III	IV3.L10	94	100	218	236	158	161
III	IV3.L11	90	96	218	218	158	158
III	IV3.L12	90	96	218	218	158	161
III	IV3.L13	94	100	218	218	158	161
IV	IV3.L14	90	94	218	236	158	161
IV	IV3.L15	96	100	218	236	158	158
IV	IV3.L16	96	100	218	218	158	164
IV	IV3.L17	94	100	218	218	158	164
V	IV3.L18	90	94	218	236	158	158
V	IV3.L19	90	96	218	218	161	164
V	IV3.L20	90	96	218	236	158	158
V	IV3.L21	90	94	218	236	158	158
V	IV3.L22	94	100	218	218	158	158
V	IV3.L23	96	100	218	236	158	158
V	IV3.L24	90	96	218	236	158	161
V	IV3.L25	90	96	218	236	161	164

**V**	IV3.L26	90	94	218	236	158	164
**V**	IV3.L27	90	96	218	218	161	164
**V**	IV3.L28	96	100	218	218	158	164
**V**	IV3.L29	96	100	218	236	158	164
**V**	IV3.L30	94	100	218	236	158	158
**V**	IV3.L31	94	100	218	218	158	161
**V**	IV3.L32	94	100	218	236	158	158
**V**	IV3.L33	94	100	218	236	158	161
**V**	IV3.L34	90	96	218	218	158	161
**V**	IV3.L35	90	94	218	218	158	158
**V**	IV3.L36	96	100	218	218	158	164
**V**	IV3.L37	96	100	218	236	161	164
**V**	IV3.L38	90	96	218	218	158	161
**VI**	IV3.L39	90	94	218	236	158	164
**VI**	IV3.L40	90	94	218	218	158	164
**VI**	IV3.L41	94	100	218	218	161	164
**VI**	IV3.L42	96	100	218	218	158	161
**VI**	IV3.L43	94	100	218	236	158	164
**VI**	IV3.L44	96	100	218	218	158	158
**VII**	IV3.L46	96	100	218	218	158	158
**VII**	IV3.L47	96	100	218	236	158	158
**VIII**	IV3.L48	90	94	218	218	158	161
**VIII**	IV3.L49	90	96	218	218	161	164
**VIII**	IV3.L50	94	100	218	218	158	161
**VIII**	IV3.L51	90	96	218	218	158	161
**IX**	IV3.L52	90	94	218	236	158	161
**IX**	IV3.L53	96	100	218	218	158	158
**X**	IV3.L54	90	94	218	236	158	164

**X**	IV3.L55	90	94	218	236	158	164
**XI**	IV3.L56	90	94	218	218	158	161
**XI**	IV3.L57	96	100	218	236	158	158
**XI**	IV3.L58	96	100	218	236	161	164
**XI**	IV3.L59	90	94	218	218	161	164
**XI**	IV3.L60	94	100	218	218	158	158
**XI**	IV3.L61	90	94	218	218	158	164
**XI**	IV3.L62	90	96	218	218	161	164
**XI**	IV3.L63	96	100	218	236	158	164
**XI**	IV3.L64	94	100	218	236	158	161
**XII**	IV3.L65	90	96	218	218	158	161
**XII**	IV3.L66	90	96	218	218	158	161
**XII**	IV3.L67	90	96	218	218	158	161
**XII**	IV3.L68	90	94	218	218	158	158
**XII**	IV3.L69	90	96	218	236	158	158
**XII**	IV3.L70	94	100	218	218	158	164
**XII**	IV3.L71	90	94	218	236	158	158
**XII**	IV3.L72	90	94	218	218	158	158
**XII**	IV3.L73	90	96	218	236	158	161
**XIII**	IV3.L74	90	96	218	236	161	164
**XIII**	IV3.L75	94	100	218	218	158	164
**XIV**	IV3.L76	90	94	218	218	158	161
**XIV**	IV3.L77	90	94	218	218	158	161
**XIV**	IV3.L78	94	100	218	218	161	164
**XIV**	IV3.L79	96	100	218	236	158	161
**XIV**	IV3.L80	90	96	218	236	158	161
**XIV**	IV3.L81	94	100	218	218	158	164

Table S4. The progeny genotypes from the five most informative loci of experiment 2 (ordered by oviposition event)

Event	Sample ID	RPW02		RPW39		RPW06		RPW32		RPW38	
I	IV4.L01	300	300	193	205	78	98	218	238	157	163
I	IV4.L02	300	300	203	205	78	98	238	238	157	163
I	IV4.L03	300	300	203	205	78	98	218	238	163	163
I	IV4.L04	300	300	193	205	78	98	238	238	157	163
I	IV4.L05	300	300	203	205	78	78	238	238	157	163
I	IV4.L06	300	300	203	205	78	78	218	238	163	163
I	IV4.L07	300	300	203	205	78	98	218	238	157	163
I	IV4.L08	300	300	193	205	78	98	218	238	163	163
II	IV4.L09	300	300	193	205	98	98	238	238	163	163
II	IV4.L10	300	300	203	205	78	98	238	238	157	163
II	IV4.L11	300	300	193	205	98	98	238	238	163	163
II	IV4.L12	300	300	203	205	78	78	238	238	157	163
II	IV4.L13	300	300	203	205	98	98	218	218	157	163
II	IV4.L14	300	300	193	205	78	98	218	238	157	163
II	IV4.L15	300	300	203	205	98	98	238	238	157	163
II	IV4.L16	300	300	203	205	78	98	218	238	157	163
II	IV4.L17	300	300	193	205	78	98	218	218	157	163
II	IV4.L18	300	300	203	205	78	98	218	238	157	163
II	IV4.L19	300	300	203	205	78	98	218	238	163	163
II	IV4.L20	300	300	203	205	78	98	218	238	163	163
II	IV4.L21	300	300	203	205	78	98	218	238	163	163
II	IV4.L22	300	300	193	205	78	98	218	238	157	163
II	IV4.L23	300	300	203	205	78	78	238	238	163	163
II	IV4.L24	300	300	203	205	78	98	218	218	157	163
II	IV4.L25	300	300	193	205	78	98	218	218	163	163

**II**	**IV4.L26**	300	300	203	205	78	78	218	238	163	163
**II**	**IV4.L27**	300	300	203	205	98	98	238	238	163	163
**II**	**IV4.L28**	300	300	193	205	78	78	218	218	157	163
**II**	**IV4.L29**	300	300	193	205	78	98	238	238	157	163
**II**	**IV4.L30**	300	300	203	205	78	98	218	238	163	163
**II**	**IV4.L31**	300	300	203	205	78	98	218	238	157	163
**II**	**IV4.L32**	300	300	203	205	78	78	218	238	157	163
**II**	**IV4.L33**	300	300	193	205	78	98	238	238	157	163
**II**	**IV4.L34**	300	300	203	205	78	98	218	218	163	163
**II**	**IV4.L35**	300	300	193	205	78	78	218	238	157	163
**II**	**IV4.L36**	300	300	203	205	98	98	238	238	163	163
**II**	**IV4.L37**	300	300	203	205	98	98	218	218	157	163
**II**	**IV4.L38**	300	300	203	205	78	98	218	218	157	163
**III**	**IV4.L39**	300	304	185	193	78	98	236	238	163	163
**III**	**IV4.L40**	300	300	203	205	78	98	218	238	157	163
**III**	**IV4.L41**	300	300	193	205	78	78	218	238	157	163
**III**	**IV4.L42**	300	300	193	205	78	78	218	218	157	163
**III**	**IV4.L43**	300	300	193	205	98	98	218	218	163	163
**III**	**IV4.L44**	300	300	193	205	78	98	218	218	163	163
**III**	**IV4.L45**	300	300	203	205	78	98	218	238	157	163
**III**	**IV4.L46**	300	300	193	205	78	78	238	238	163	163
**III**	**IV4.L47**	300	300	203	205	78	98	238	238	163	163
**IV**	**IV4.L48**	300	300	203	205	78	98	218	238	157	163
**IV**	**IV4.L49**	300	300	193	205	78	98	218	218	163	163
**IV**	**IV4.L50**	300	300	203	205	78	98	218	238	157	163
**IV**	**IV4.L51**	300	300	193	205	78	78	238	238	163	163
**IV**	**IV4.L52**	300	300	193	205	98	98	218	218	163	163
**IV**	**IV4.L53**	300	300	203	205	78	98	218	238	163	163

**V**	**IV4.L54**	300	300	193	205	98	98	218	238	163	163
**V**	**IV4.L55**	300	300	203	205	78	98	218	218	163	163
**V**	**IV4.L56**	300	300	203	205	78	98	238	238	163	163
**V**	**IV4.L57**	300	300	193	205	78	98	218	218	157	163
**VI**	**IV4.L58**	300	300	193	205	78	98	218	238	157	163
**VII**	**IV4.L59**	300	300	193	205	78	78	238	238	163	163
**VII**	**IV4.L60**	300	300	203	205	98	98	238	238	163	163
**VII**	**IV4.L61**	300	300	193	205	78	98	218	218	163	163
**VII**	**IV4.L62**	300	300	203	205	78	98	218	238	157	163
**VII**	**IV4.L63**	300	300	203	205	78	98	238	238	163	163
**VII**	**IV4.L64**	300	300	203	205	78	98	218	238	163	163
**VII**	**IV4.L65**	300	300	193	205	98	98	238	238	157	163
**VII**	**IV4.L66**	300	300	193	205	78	98	218	218	163	163
**VII**	**IV4.L67**	300	300	203	205	98	98	218	218	157	163
**VII**	**IV4.L68**	300	300	193	205	98	98	218	238	163	163
**VII**	**IV4.L69**	300	300	193	205	78	98	218	218	163	163
**VII**	**IV4.L70**	300	300	203	205	78	78	218	238	163	163
**VII**	**IV4.L71**	300	300	193	205	78	98	218	218	157	163
**VIII**	**IV4.L72**	300	300	193	205	78	98	218	238	157	163
**VIII**	**IV4.L73**	300	300	193	205	98	98	218	218	157	163
**VIII**	**IV4.L74**	300	300	203	205	98	98	218	238	157	163
**VIII**	**IV4.L75**	300	300	193	205	78	78	218	218	163	163
**VIII**	**IV4.L76**	300	300	203	205	78	78	218	238	163	163
**VIII**	**IV4.L77**	300	300	203	205	98	98	218	238	163	163
**VIII**	**IV4.L78**	300	300	203	205	78	78	218	238	163	163
**X**	**IV4.L79**	300	300	203	205	78	98	238	238	157	163
**X**	**IV4.L80**	300	300	203	205	78	98	218	238	157	163
**X**	**IV4.L81**	300	300	203	205	78	98	218	238	163	163

**XI**	**IV4.L82**	300	300	193	205	78	98	218	238	163	163
**XI**	**IV4.L83**	300	300	193	205	78	98	238	238	157	163
**XII**	**IV4.L84**	300	300	193	205	78	78	238	238	163	163
**XII**	**IV4.L85**	300	312	185	203	78	98	218	218	163	165
**XII**	**IV4.L86**	300	300	193	205	78	98	238	238	157	163
**XII**	**IV4.L87**	300	300	193	205	78	98	218	218	163	163
**XII**	**IV4.L88**	300	300	203	205	78	78	218	238	163	163
**XII**	**IV4.L89**	300	300	193	205	78	98	218	218	157	163
**XII**	**IV4.L90**	300	300	193	205	78	98	238	238	157	163
**XII**	**IV4.L91**	300	300	193	205	78	78	218	218	163	163
**XII**	**IV4.L92**	300	300	203	205	78	98	238	238	163	163
**XII**	**IV4.L93**	300	300	203	205	78	78	218	238	163	163
**XII**	**IV4.L94**	300	300	193	205	78	98	238	238	157	163
**XII**	**IV4.L95**	300	300	193	205	78	78	238	238	157	163
**XII**	**IV4.L96**	300	300	193	205	78	98	218	218	163	163
**XII**	**IV4.L97**	300	300	203	205	78	98	218	238	157	163
**XII**	**IV4.L98**	300	300	193	205	78	98	218	238	163	163
**XII**	**IV4.L99**	300	300	193	205	78	78	238	238	163	163
**XII**	**IV4.L100**	300	300	193	205	78	98	238	238	163	163
**XII**	**IV4.L101**	300	300	203	205	78	78	238	238	163	163
**XII**	**IV4.L102**	300	300	203	205	78	98	218	238	163	163
**XIII**	**IV4.L103**	300	300	203	205	78	78	218	238	163	163
**XIII**	**IV4.L104**	300	300	203	205	78	78	218	218	163	163
**XIII**	**IV4.L105**	300	300	193	205	78	98	238	238	157	163
**XIII**	**IV4.L106**	300	300	193	205	78	98	238	238	163	163
**XIV**	**IV4.L107**	300	300	203	205	78	98	238	238	163	163
**XIV**	**IV4.L108**	300	300	193	205	78	78	218	218	157	163
**XV**	**IV4.L109**	300	300	193	205	78	98	238	238	163	163

**XV**	**IV4.L110**	300	300	193	205	78	98	218	218	157	163
**XV**	**IV4.L111**	300	300	193	205	78	78	218	238	163	163
**XV**	**IV4.L112**	300	300	193	205	78	98	218	218	157	163
**XV**	**IV4.L113**	300	300	193	205	78	98	238	238	163	163
**XV**	**IV4.L114**	300	300	203	205	78	98	218	218	157	163
**XV**	**IV4.L115**	300	300	203	205	98	98	238	238	163	163
**XV**	**IV4.L116**	300	300	193	205	78	98	218	218	163	163
**XV**	**IV4.L117**	0	0	203	205	78	98	218	218	163	163
**XV**	**IV4.L118**	300	300	193	205	78	98	218	218	163	163
**XVI**	**IV4.L119**	300	300	193	205	98	98	218	238	157	163
**XVI**	**IV4.L120**	300	300	203	205	78	98	218	238	157	163
**XVI**	**IV4.L121**	300	300	193	205	78	78	218	238	163	163
**XVI**	**IV4.L122**	300	300	193	205	78	78	238	238	157	163
**XVI**	**IV4.L123**	300	300	193	205	78	78	218	218	157	163
**XVII**	**IV4.L124**	300	300	193	205	98	98	218	238	157	163
**XVIII**	**IV4.L125**	300	300	203	205	78	78	218	238	163	163

Table S5. The paternity assignment of experiment #1 using three microsatellite loci and Cervus v.3.0.6 software

Offspring	NE-2P	Candidate father ID	Trio loci compared	Trio loci mismatching	Trio LOD score	Trio confidence
IV3.L01	5.93E-003	M2	3	0	4.56E+000	*
IV3.L02	5.93E-003	M2	3	0	4.56E+000	*
IV3.L03	1.50E-002	M2	3	0	3.49E+000	*
IV3.L04	2.30E-003	M2	3	0	5.78E+000	*
IV3.L05	1.22E-003	M2	3	0	6.17E+000	*
IV3.L06	7.32E-002	M2	3	0	1.89E+000	*
IV3.L07	2.44E-002	M2	3	0	2.65E+000	*
IV3.L08	1.81E-002	M2	3	0	3.32E+000	*
IV3.L09	3.73E-003	M2	3	0	4.93E+000	*
IV3.L10	3.73E-003	M2	3	0	4.93E+000	*
IV3.L11	7.32E-002	M2	3	0	1.88E+000	*
IV3.L12	1.19E-001	M2	3	0	1.04E+000	*
IV3.L13	2.44E-002	M2	3	0	2.65E+000	*
IV3.L14	1.81E-002	M2	3	0	3.33E+000	*
IV3.L15	2.30E-003	M2	3	0	5.77E+000	*
IV3.L16	1.22E-003	M2	3	0	6.17E+000	*
IV3.L17	1.22E-003	M2	3	0	6.18E+000	*
IV3.L18	1.12E-002	M2	3	0	4.17E+000	*
IV3.L19	5.93E-003	M2	3	0	4.55E+000	*
IV3.L20	1.12E-002	M2	3	0	4.16E+000	*
IV3.L21	1.12E-002	M2	3	0	4.17E+000	*
IV3.L22	1.50E-002	M2	3	0	3.49E+000	*
IV3.L23	2.30E-003	M2	3	0	5.77E+000	*
IV3.L24	1.81E-002	M2	3	0	3.32E+000	*
IV3.L25	9.07E-004	M2	3	0	6.83E+000	*
IV3.L26	9.07E-004	M2	3	0	6.85E+000	*
IV3.L27	5.93E-003	M2	3	0	4.55E+000	*
IV3.L28	1.22E-003	M2	3	0	6.17E+000	*
IV3.L29	1.86E-004	M2	3	0	8.45E+000	*
IV3.L30	2.30E-003	M2	3	0	5.78E+000	*

Offspring	NE-2P	Candidate father ID	Trio loci compared	Trio loci mismatching	Trio LOD score	Trio confidence
IV3.L31	2.44E-002	M2	3	0	2.65E+000	*
IV3.L32	2.30E-003	M2	3	0	5.78E+000	*
IV3.L33	3.73E-003	M2	3	0	4.93E+000	*
IV3.L34	1.19E-001	M2	3	0	1.04E+000	*
IV3.L35	7.32E-002	M2	3	0	1.89E+000	*
IV3.L36	1.22E-003	M2	3	0	6.17E+000	*
IV3.L37	1.86E-004	M2	3	0	8.44E+000	*
IV3.L38	1.19E-001	M2	3	0	1.04E+000	*
IV3.L39	9.07E-004	M2	3	0	6.85E+000	*
IV3.L40	5.93E-003	M2	3	0	4.57E+000	*
IV3.L41	1.22E-003	M2	3	0	6.17E+000	*
IV3.L42	2.44E-002	M2	3	0	2.65E+000	*
IV3.L43	1.86E-004	M2	3	0	8.46E+000	*
IV3.L44	1.50E-002	M2	3	0	3.49E+000	*
IV3.L45	5.93E-003	M2	3	0	4.56E+000	*
IV3.L46	1.50E-002	M2	3	0	3.49E+000	*
IV3.L47	2.30E-003	M2	3	0	5.77E+000	*
IV3.L48	1.19E-001	M2	3	0	1.04E+000	*
IV3.L49	5.93E-003	M2	3	0	4.55E+000	*
IV3.L50	2.44E-002	M2	3	0	2.65E+000	*
IV3.L51	1.19E-001	M2	3	0	1.04E+000	*
IV3.L52	1.81E-002	M2	3	0	3.33E+000	*
IV3.L53	1.50E-002	M2	3	0	3.49E+000	*
IV3.L54	9.07E-004	M2	3	0	6.85E+000	*
IV3.L55	9.07E-004	M2	3	0	6.85E+000	*
IV3.L56	1.19E-001	M2	3	0	1.04E+000	*
IV3.L57	2.30E-003	M2	3	0	5.77E+000	*
IV3.L58	1.86E-004	M2	3	0	8.44E+000	*
IV3.L59	5.93E-003	M2	3	0	4.56E+000	*
IV3.L60	1.50E-002	M2	3	0	3.49E+000	*
IV3.L61	5.93E-003	M2	3	0	4.57E+000	*
IV3.L62	5.93E-003	M2	3	0	4.55E+000	*

Offspring	NE-2P	Candidate father ID	Trio loci compared	Trio loci mismatching	Trio LOD score	Trio confidence
IV3.L63	1.86E-004	M2	3	0	8.45E+000	*
IV3.L64	3.73E-003	M2	3	0	4.93E+000	*
IV3.L65	1.19E-001	M2	3	0	1.04E+000	*
IV3.L66	1.19E-001	M2	3	0	1.04E+000	*
IV3.L67	1.19E-001	M2	3	0	1.04E+000	*
IV3.L68	7.32E-002	M2	3	0	1.89E+000	*
IV3.L69	1.12E-002	M2	3	0	4.16E+000	*
IV3.L70	1.22E-003	M2	3	0	6.18E+000	*
IV3.L71	1.12E-002	M2	3	0	4.17E+000	*
IV3.L72	7.32E-002	M2	3	0	1.89E+000	*
IV3.L73	1.81E-002	M2	3	0	3.32E+000	*
IV3.L74	9.07E-004	M2	3	0	6.83E+000	*
IV3.L75	1.22E-003	M2	3	0	6.18E+000	*
IV3.L76	1.19E-001	M2	3	0	1.04E+000	*
IV3.L77	1.19E-001	M2	3	0	1.04E+000	*
IV3.L78	1.22E-003	M2	3	0	6.17E+000	*
IV3.L79	3.73E-003	M2	3	0	4.93E+000	*
IV3.L80	1.81E-002	M2	3	0	3.32E+000	*
IV3.L81	1.22E-003	M2	3	0	6.18E+000	*

NE-2P: second parent non-exclusion probability (first parent kwown); **Trio LOD score**: Log-likelihood ratio for a parents-offspring relationship between the kown mother, the first candidate father and the offspring; *****: for strict confidence.

Table S6. The paternity assignment of experiment #2 using five microsatellite loci and Cervus v.3.0.6 software

Offspring	NE-2P	Candidate father ID	Trio loci compared	Trio loci mismatching	Trio LOD score	Trio confidence
IV4.L01	8.90E-002	M2	5	0	3.74E+000	*
IV4.L02	2.86E-002	M2	5	0	4.70E+000	*
IV4.L03	1.56E-001	M2	5	0	3.10E+000	*
IV4.L04	2.86E-002	M2	5	0	4.70E+000	*
IV4.L05	2.34E-002	M2	5	0	4.27E+000	*
IV4.L06	1.28E-001	M2	5	0	2.66E+000	*
IV4.L07	8.90E-002	M2	5	0	3.74E+000	*
IV4.L08	1.56E-001	M2	5	0	3.09E+000	*
IV4.L09	1.36E-002	M2	5	0	4.85E+000	*
IV4.L10	2.86E-002	M2	5	0	4.70E+000	*
IV4.L11	1.36E-002	M2	5	0	4.85E+000	*
IV4.L12	2.34E-002	M2	5	0	4.27E+000	*
IV4.L13	2.27E-002	M2	5	0	4.06E+000	*
IV4.L14	8.90E-002	M2	5	0	3.74E+000	*
IV4.L15	7.74E-003	M2	5	0	5.49E+000	*
IV4.L16	8.90E-002	M2	5	0	3.74E+000	*
IV4.L17	8.39E-002	M2	5	0	3.27E+000	*
IV4.L18	8.90E-002	M2	5	0	3.74E+000	*
IV4.L19	1.56E-001	M2	5	0	3.10E+000	*
IV4.L20	1.56E-001	M2	5	0	3.10E+000	*
IV4.L21	1.56E-001	M2	5	0	3.10E+000	*
IV4.L22	8.90E-002	M2	5	0	3.74E+000	*
IV4.L23	4.11E-002	M2	5	0	3.62E+000	*
IV4.L24	8.39E-002	M2	5	0	3.27E+000	*
IV4.L25	1.47E-001	M2	5	0	2.62E+000	*

Offspring	NE-2P	Candidate father ID	Trio loci compared	Trio loci mismatching	Trio LOD score	Trio confidence
IV4.L26	1.28E-001	M2	5	0	2.66E+000	*
IV4.L27	1.36E-002	M2	5	0	4.85E+000	*
IV4.L28	6.87E-002	M2	5	0	2.83E+000	*
IV4.L29	2.86E-002	M2	5	0	4.70E+000	*
IV4.L30	1.56E-001	M2	5	0	3.10E+000	*
IV4.L31	8.90E-002	M2	5	0	3.74E+000	*
IV4.L32	7.29E-002	M2	5	0	3.31E+000	*
IV4.L33	2.86E-002	M2	5	0	4.70E+000	*
IV4.L34	1.47E-001	M2	5	0	2.62E+000	*
IV4.L35	7.29E-002	M2	5	0	3.30E+000	*
IV4.L36	1.36E-002	M2	5	0	4.85E+000	*
IV4.L37	2.27E-002	M2	5	0	4.06E+000	*
IV4.L38	8.39E-002	M2	5	0	3.27E+000	*
IV4.L39	1.69E-003	M1	5	0	7.14E+000	*
IV4.L40	8.90E-002	M2	5	0	3.74E+000	*
IV4.L41	7.29E-002	M2	5	0	3.30E+000	*
IV4.L42	6.87E-002	M2	5	0	2.83E+000	*
IV4.L43	3.99E-002	M2	5	0	3.41E+000	*
IV4.L44	1.47E-001	M2	5	0	2.62E+000	*
IV4.L45	8.90E-002	M2	5	0	3.74E+000	*
IV4.L46	4.11E-002	M2	5	0	3.62E+000	*
IV4.L47	5.02E-002	M2	5	0	4.06E+000	*
IV4.L48	8.90E-002	M2	5	0	3.74E+000	*
IV4.L49	1.47E-001	M2	5	0	2.62E+000	*
IV4.L50	8.90E-002	M2	5	0	3.74E+000	*
IV4.L51	4.11E-002	M2	5	0	3.62E+000	*
IV4.L52	3.99E-002	M2	5	0	3.41E+000	*

Offspring	NE-2P	Candidate father ID	Trio loci compared	Trio loci mismatching	Trio LOD score	Trio confidence
IV4.L53	1.56E-001	M2	5	0	3.10E+000	*
IV4.L54	4.23E-002	M2	5	0	3.89E+000	*
IV4.L55	1.47E-001	M2	5	0	2.62E+000	*
IV4.L56	5.02E-002	M2	5	0	4.06E+000	*
IV4.L57	8.39E-002	M2	5	0	3.27E+000	*
IV4.L58	8.90E-002	M2	5	0	3.74E+000	*
IV4.L59	4.11E-002	M2	5	0	3.62E+000	*
IV4.L60	1.36E-002	M2	5	0	4.85E+000	*
IV4.L61	1.47E-001	M2	5	0	2.62E+000	*
IV4.L62	8.90E-002	M2	5	0	3.74E+000	*
IV4.L63	5.02E-002	M2	5	0	4.06E+000	*
IV4.L64	1.56E-001	M2	5	0	3.10E+000	*
IV4.L65	7.74E-003	M2	5	0	5.49E+000	*
IV4.L66	1.47E-001	M2	5	0	2.62E+000	*
IV4.L67	2.27E-002	M2	5	0	4.06E+000	*
IV4.L68	4.23E-002	M2	5	0	3.89E+000	*
IV4.L69	1.47E-001	M2	5	0	2.62E+000	*
IV4.L70	1.28E-001	M2	5	0	2.66E+000	*
IV4.L71	8.39E-002	M2	5	0	3.27E+000	*
IV4.L72	8.90E-002	M2	5	0	3.74E+000	*
IV4.L73	2.27E-002	M2	5	0	4.06E+000	*
IV4.L74	2.41E-002	M2	5	0	4.53E+000	*
IV4.L75	1.21E-001	M2	5	0	2.18E+000	*
IV4.L76	1.28E-001	M2	5	0	2.66E+000	*
IV4.L77	4.23E-002	M2	5	0	3.89E+000	*
IV4.L78	1.28E-001	M2	5	0	2.66E+000	*
IV4.L79	2.86E-002	M2	5	0	4.70E+000	*

Offspring	NE-2P	Candidate father ID	Trio loci compared	Trio loci mismatching	Trio LOD score	Trio confidence
IV4.L80	8.90E-002	M2	5	0	3.74E+000	*
IV4.L81	1.56E-001	M2	5	0	3.10E+000	*
IV4.L82	1.56E-001	M2	5	0	3.09E+000	*
IV4.L83	2.86E-002	M2	5	0	4.70E+000	*
IV4.L84	4.11E-002	M2	5	0	3.62E+000	*
IV4.L85	1.77E-003	M1	5	0	6.83E+000	*
IV4.L86	2.86E-002	M2	5	0	4.70E+000	*
IV4.L87	1.47E-001	M2	5	0	2.62E+000	*
IV4.L88	1.28E-001	M2	5	0	2.66E+000	*
IV4.L89	8.39E-002	M2	5	0	3.27E+000	*
IV4.L90	2.86E-002	M2	5	0	4.70E+000	*
IV4.L91	1.21E-001	M2	5	0	2.18E+000	*
IV4.L92	5.02E-002	M2	5	0	4.06E+000	*
IV4.L93	1.28E-001	M2	5	0	2.66E+000	*
IV4.L94	2.86E-002	M2	5	0	4.70E+000	*
IV4.L95	2.34E-002	M2	5	0	4.26E+000	*
IV4.L96	1.47E-001	M2	5	0	2.62E+000	*
IV4.L97	8.90E-002	M2	5	0	3.74E+000	*
IV4.L98	1.56E-001	M2	5	0	3.09E+000	*
IV4.L99	4.11E-002	M2	5	0	3.62E+000	*
IV4.L100	5.02E-002	M2	5	0	4.06E+000	*
IV4.L101	4.11E-002	M2	5	0	3.62E+000	*
IV4.L102	1.56E-001	M2	5	0	3.10E+000	*
IV4.L103	1.28E-001	M2	5	0	2.66E+000	*
IV4.L104	1.21E-001	M2	5	0	2.19E+000	*
IV4.L105	2.86E-002	M2	5	0	4.70E+000	*
IV4.L106	5.02E-002	M2	5	0	4.06E+000	*

Offspring	NE-2P	Candidate father ID	Trio loci compared	Trio loci mismatching	Trio LOD score	Trio confidence
IV4.L107	5.02E-002	M2	5	0	4.06E+000	*
IV4.L108	6.87E-002	M2	5	0	2.83E+000	*
IV4.L109	5.02E-002	M2	5	0	4.06E+000	*
IV4.L110	8.39E-002	M2	5	0	3.27E+000	*
IV4.L111	1.28E-001	M2	5	0	2.66E+000	*
IV4.L112	8.39E-002	M2	5	0	3.27E+000	*
IV4.L113	5.02E-002	M2	5	0	4.06E+000	*
IV4.L114	8.39E-002	M2	5	0	3.27E+000	*
IV4.L115	1.36E-002	M2	5	0	4.85E+000	*
IV4.L116	1.47E-001	M2	5	0	2.62E+000	*
IV4.L117	2.04E-001	M2	4	0	1.88E+000	*
IV4.L118	1.47E-001	M2	5	0	2.62E+000	*
IV4.L119	2.41E-002	M2	5	0	4.53E+000	*
IV4.L120	8.90E-002	M2	5	0	3.74E+000	*
IV4.L121	1.28E-001	M2	5	0	2.66E+000	*
IV4.L122	2.34E-002	M2	5	0	4.26E+000	*
IV4.L123	6.87E-002	M2	5	0	2.83E+000	*
IV4.L124	2.41E-002	M2	5	0	4.53E+000	*
IV4.L125	1.28E-001	M2	5	0	2.66E+000	*

NE-2P: second parent non-exclusion probability (first parent kwown); **Trio LOD score**: Log-likelihood ratio for a parents-offspring relationship between the kown mother, the first candidate father and the offspring; *: for strict confidence.

Table S7. The paternity assignment of experiment #1 using Patri software

Number of breeding males = 2

expected numbers of sampled offspring from sampled fathers: 81

non-zero probabilities of paternity for each mother-offspring pair:

**labels:**		**group:**	**P(father)**
calf:	IV3.L01		
mother:	IV3.F1		
male:	IV3.M2	0	1.000000E+00
	0	1.000000E+00	
	all	1.000000E+00	
calf:	IV3.L02		
mother:	IV3.F1		
male:	IV3.M2	0	1.000000E+00
	0	1.000000E+00	
	all	1.000000E+00	
calf:	IV3.L03		
mother:	IV3.F1		
male:	IV3.M2	0	1.000000E+00
	0	1.000000E+00	
	all	1.000000E+00	
calf:	IV3.L04		
mother:	IV3.F1		
male:	IV3.M2	0	1.000000E+00
	0	1.000000E+00	
	all	1.000000E+00	
calf:	IV3.L05		
mother:	IV3.F1		
male:	IV3.M2	0	1.000000E+00
	0	1.000000E+00	
	all	1.000000E+00	
calf:	IV3.L06		
mother:	IV3.F1		
male:	IV3.M2	0	1.000000E+00
	0	1.000000E+00	
	all	1.000000E+00	
calf:	IV3.L07		
mother:	IV3.F1		
male:	IV3.M2	0	1.000000E+00
	0	1.000000E+00	
	all	1.000000E+00	

**labels:**		**group:**	**P(father)**
calf:	IV3.L08		
mother:	IV3.F1		
male:	IV3.M2	0	1.000000E+00
	0	1.000000E+00	
	all	1.000000E+00	
calf:	IV3.L09		
mother:	IV3.F1		
male:	IV3.M2	0	1.000000E+00
	0	1.000000E+00	
	all	1.000000E+00	
calf:	IV3.L10		
mother:	IV3.F1		
male:	IV3.M2	0	1.000000E+00
	0	1.000000E+00	
	all	1.000000E+00	
calf:	IV3.L11		
mother:	IV3.F1		
male:	IV3.M2	0	1.000000E+00
	0	1.000000E+00	
	all	1.000000E+00	
calf:	IV3.L12		
mother:	IV3.F1		
male:	IV3.M2	0	1.000000E+00
	0	1.000000E+00	
	all	1.000000E+00	
calf:	IV3.L13		
mother:	IV3.F1		
male:	IV3.M2	0	1.000000E+00
	0	1.000000E+00	
	all	1.000000E+00	
calf:	IV3.L14		
mother:	IV3.F1		
male:	IV3.M2	0	1.000000E+00
	0	1.000000E+00	
	all	1.000000E+00	

**labels:**		**group:**	**P(father)**
calf:	IV3.L15		
mother:	IV3.F1		
male:	IV3.M2	0	1.000000E+00
		0	1.000000E+00
		all	1.000000E+00
calf:	IV3.L16		
mother:	IV3.F1		
male:	IV3.M2	0	1.000000E+00
		0	1.000000E+00
		all	1.000000E+00
calf:	IV3.L17		
mother:	IV3.F1		
male:	IV3.M2	0	1.000000E+00
		0	1.000000E+00
		all	1.000000E+00
calf:	IV3.L18		
mother:	IV3.F1		
male:	IV3.M2	0	1.000000E+00
		0	1.000000E+00
		all	1.000000E+00
calf:	IV3.L19		
mother:	IV3.F1		
male:	IV3.M2	0	1.000000E+00
		0	1.000000E+00
		all	1.000000E+00
calf:	IV3.L20		
mother:	IV3.F1		
male:	IV3.M2	0	1.000000E+00
		0	1.000000E+00
		all	1.000000E+00
calf:	IV3.L21		
mother:	IV3.F1		
male:	IV3.M2	0	1.000000E+00
		0	1.000000E+00
		all	1.000000E+00
calf:	IV3.L22		
mother:	IV3.F1		
male:	IV3.M2	0	1.000000E+00
		0	1.000000E+00
		all	1.000000E+00
calf:	IV3.L23		
mother:	IV3.F1		
male:	IV3.M2	0	1.000000E+00
		0	1.000000E+00
		all	1.000000E+00
calf:	IV3.L24		
mother:	IV3.F1		
male:	IV3.M2	0	1.000000E+00
		0	1.000000E+00
		all	1.000000E+00

**labels:**		**group:**	**P(father)**
calf:	IV3.L25		
mother:	IV3.F1		
male:	IV3.M2	0	1.000000E+00
		0	1.000000E+00
		all	1.000000E+00
calf:	IV3.L26		
mother:	IV3.F1		
male:	IV3.M2	0	1.000000E+00
		0	1.000000E+00
		all	1.000000E+00
calf:	IV3.L27		
mother:	IV3.F1		
male:	IV3.M2	0	1.000000E+00
		0	1.000000E+00
		all	1.000000E+00
calf:	IV3.L28		
mother:	IV3.F1		
male:	IV3.M2	0	1.000000E+00
		0	1.000000E+00
		all	1.000000E+00
calf:	IV3.L29		
mother:	IV3.F1		
male:	IV3.M2	0	1.000000E+00
		0	1.000000E+00
		all	1.000000E+00
calf:	IV3.L30		
mother:	IV3.F1		
male:	IV3.M2	0	1.000000E+00
		0	1.000000E+00
		all	1.000000E+00
calf:	IV3.L31		
mother:	IV3.F1		
male:	IV3.M2	0	1.000000E+00
		0	1.000000E+00
		all	1.000000E+00
calf:	IV3.L32		
mother:	IV3.F1		
male:	IV3.M2	0	1.000000E+00
		0	1.000000E+00
		all	1.000000E+00
calf:	IV3.L33		
mother:	IV3.F1		
male:	IV3.M2	0	1.000000E+00
		0	1.000000E+00
		all	1.000000E+00
calf:	IV3.L34		
mother:	IV3.F1		
male:	IV3.M2	0	1.000000E+00
		0	1.000000E+00
		all	1.000000E+00

**labels:**			**group:**	**P(father)**
calf:	IV3.L35			
mother:	IV3.F1			
male:	IV3.M2		0	1.000000E+00
		0	1.000000E+00	
		all	1.000000E+00	
calf:	IV3.L36			
mother:	IV3.F1			
male:	IV3.M2		0	1.000000E+00
		0	1.000000E+00	
		all	1.000000E+00	
calf:	IV3.L37			
mother:	IV3.F1			
male:	IV3.M2		0	1.000000E+00
		0	1.000000E+00	
		all	1.000000E+00	
calf:	IV3.L38			
mother:	IV3.F1			
male:	IV3.M2		0	1.000000E+00
		0	1.000000E+00	
		all	1.000000E+00	
calf:	IV3.L39			
mother:	IV3.F1			
male:	IV3.M2		0	1.000000E+00
		0	1.000000E+00	
		all	1.000000E+00	
calf:	IV3.L40			
mother:	IV3.F1			
male:	IV3.M2		0	1.000000E+00
		0	1.000000E+00	
		all	1.000000E+00	
calf:	IV3.L41			
mother:	IV3.F1			
male:	IV3.M2		0	1.000000E+00
		0	1.000000E+00	
		all	1.000000E+00	
calf:	IV3.L42			
mother:	IV3.F1			
male:	IV3.M2		0	1.000000E+00
		0	1.000000E+00	
		all	1.000000E+00	
calf:	IV3.L43			
mother:	IV3.F1			
male:	IV3.M2		0	1.000000E+00
		0	1.000000E+00	
		all	1.000000E+00	
calf:	IV3.L44			
mother:	IV3.F1			
male:	IV3.M2		0	1.000000E+00
		0	1.000000E+00	
		all	1.000000E+00	

**labels:**			**group:**	**P(father)**
calf:	IV3.L45			
mother:	IV3.F1			
male:	IV3.M2		0	1.000000E+00
		0	1.000000E+00	
		all	1.000000E+00	
calf:	IV3.L46			
mother:	IV3.F1			
male:	IV3.M2		0	1.000000E+00
		0	1.000000E+00	
		all	1.000000E+00	
calf:	IV3.L47			
mother:	IV3.F1			
male:	IV3.M2		0	1.000000E+00
		0	1.000000E+00	
		all	1.000000E+00	
calf:	IV3.L48			
mother:	IV3.F1			
male:	IV3.M2		0	1.000000E+00
		0	1.000000E+00	
		all	1.000000E+00	
calf:	IV3.L49			
mother:	IV3.F1			
male:	IV3.M2		0	1.000000E+00
		0	1.000000E+00	
		all	1.000000E+00	
calf:	IV3.L50			
mother:	IV3.F1			
male:	IV3.M2		0	1.000000E+00
		0	1.000000E+00	
		all	1.000000E+00	
calf:	IV3.L51			
mother:	IV3.F1			
male:	IV3.M2		0	1.000000E+00
		0	1.000000E+00	
		all	1.000000E+00	
calf:	IV3.L52			
mother:	IV3.F1			
male:	IV3.M2		0	1.000000E+00
		0	1.000000E+00	
		all	1.000000E+00	
calf:	IV3.L53			
mother:	IV3.F1			
male:	IV3.M2		0	1.000000E+00
		0	1.000000E+00	
		all	1.000000E+00	
calf:	IV3.L54			
mother:	IV3.F1			
male:	IV3.M2		0	1.000000E+00
		0	1.000000E+00	
		all	1.000000E+00	

labels:			group:	P(father)
calf:	IV3.L55			
mother:	IV3.F1			
male:	IV3.M2		0	1.000000E+00
		0	1.000000E+00	
		all	1.000000E+00	
calf:	IV3.L56			
mother:	IV3.F1			
male:	IV3.M2		0	1.000000E+00
		0	1.000000E+00	
		all	1.000000E+00	
calf:	IV3.L57			
mother:	IV3.F1			
male:	IV3.M2		0	1.000000E+00
		0	1.000000E+00	
		all	1.000000E+00	
calf:	IV3.L58			
mother:	IV3.F1			
male:	IV3.M2		0	1.000000E+00
		0	1.000000E+00	
		all	1.000000E+00	
calf:	IV3.L59			
mother:	IV3.F1			
male:	IV3.M2		0	1.000000E+00
		0	1.000000E+00	
		all	1.000000E+00	
calf:	IV3.L60			
mother:	IV3.F1			
male:	IV3.M2		0	1.000000E+00
		0	1.000000E+00	
		all	1.000000E+00	
calf:	IV3.L61			
mother:	IV3.F1			
male:	IV3.M2		0	1.000000E+00
		0	1.000000E+00	
		all	1.000000E+00	
calf:	IV3.L62			
mother:	IV3.F1			
male:	IV3.M2		0	1.000000E+00
		0	1.000000E+00	
		all	1.000000E+00	
calf:	IV3.L63			
mother:	IV3.F1			
male:	IV3.M2		0	1.000000E+00
		0	1.000000E+00	
		all	1.000000E+00	
calf:	IV3.L64			
mother:	IV3.F1			
male:	IV3.M2		0	1.000000E+00
		0	1.000000E+00	
		all	1.000000E+00	

labels:			group:	P(father)
calf:	IV3.L65			
mother:	IV3.F1			
male:	IV3.M2		0	1.000000E+00
		0	1.000000E+00	
		all	1.000000E+00	
calf:	IV3.L66			
mother:	IV3.F1			
male:	IV3.M2		0	1.000000E+00
		0	1.000000E+00	
		all	1.000000E+00	
calf:	IV3.L67			
mother:	IV3.F1			
male:	IV3.M2		0	1.000000E+00
		0	1.000000E+00	
		all	1.000000E+00	
calf:	IV3.L68			
mother:	IV3.F1			
male:	IV3.M2		0	1.000000E+00
		0	1.000000E+00	
		all	1.000000E+00	
calf:	IV3.L69			
mother:	IV3.F1			
male:	IV3.M2		0	1.000000E+00
		0	1.000000E+00	
		all	1.000000E+00	
calf:	IV3.L70			
mother:	IV3.F1			
male:	IV3.M2		0	1.000000E+00
		0	1.000000E+00	
		all	1.000000E+00	
calf:	IV3.L71			
mother:	IV3.F1			
male:	IV3.M2		0	1.000000E+00
		0	1.000000E+00	
		all	1.000000E+00	
calf:	IV3.L72			
mother:	IV3.F1			
male:	IV3.M2		0	1.000000E+00
		0	1.000000E+00	
		all	1.000000E+00	
calf:	IV3.L73			
mother:	IV3.F1			
male:	IV3.M2		0	1.000000E+00
		0	1.000000E+00	
		all	1.000000E+00	
calf:	IV3.L74			
mother:	IV3.F1			
male:	IV3.M2		0	1.000000E+00
		0	1.000000E+00	
		all	1.000000E+00	

**labels:**		**group:**	**P(father)**
calf:	IV3.L75		
mother:	IV3.F1		
male:	IV3.M2	0	1.000000E+00
	0	1.000000E+00	
	all	1.000000E+00	
calf:	IV3.L76		
mother:	IV3.F1		
male:	IV3.M2	0	1.000000E+00
	0	1.000000E+00	
	all	1.000000E+00	
calf:	IV3.L77		
mother:	IV3.F1		
male:	IV3.M2	0	1.000000E+00
	0	1.000000E+00	
	all	1.000000E+00	
calf:	IV3.L78		
mother:	IV3.F1		
male:	IV3.M2	0	1.000000E+00
	0	1.000000E+00	
	all	1.000000E+00	
calf:	IV3.L79		
mother:	IV3.F1		
male:	IV3.M2	0	1.000000E+00
	0	1.000000E+00	
	all	1.000000E+00	
calf:	IV3.L80		
mother:	IV3.F1		
male:	IV3.M2	0	1.000000E+00
	0	1.000000E+00	
	all	1.000000E+00	
calf:	IV3.L81		
mother:	IV3.F1		
male:	IV3.M2	0	1.000000E+00
	0	1.000000E+00	
	all	1.000000E+00	

Legend:

<offspring ID>		
<mother ID>		
<male x ID>	<group of male x>	<Probability of male x is the father>
	<group y>	< Probability of male x belongs to group y>
	all	< Probability of male x has been sampled>

Table S8. The paternity assignment of experiment #2 using Patri software

Number of breeding males = 2

expected numbers of sampled offspring from sampled fathers: 125

non-zero probabilities of paternity for each mother-offspring pair:

labels:		group:	P(father)
calf:	IV4.L01		
mother:	IV4.F1		
male:	IV4.M2	0	1.000000E+00
		0	1.000000E+00
		all	1.000000E+00
calf:	IV4.L02		
mother:	IV4.F1		
male:	IV4.M2	0	1.000000E+00
		0	1.000000E+00
		all	1.000000E+00
calf:	IV4.L03		
mother:	IV4.F1		
male:	IV4.M2	0	1.000000E+00
		0	1.000000E+00
		all	1.000000E+00
calf:	IV4.L04		
mother:	IV4.F1		
male:	IV4.M2	0	1.000000E+00
		0	1.000000E+00
		all	1.000000E+00
calf:	IV4.L05		
mother:	IV4.F1		
male:	IV4.M2	0	1.000000E+00
		0	1.000000E+00
		all	1.000000E+00
calf:	IV4.L06		
mother:	IV4.F1		
male:	IV4.M2	0	1.000000E+00
		0	1.000000E+00
		all	1.000000E+00
calf:	IV4.L07		
mother:	IV4.F1		
male:	IV4.M2	0	1.000000E+00
		0	1.000000E+00
		all	1.000000E+00
calf:	IV4.L08		
mother:	IV4.F1		
male:	IV4.M2	0	1.000000E+00
		0	1.000000E+00
		all	1.000000E+00
calf:	IV4.L17		
mother:	IV4.F1		
male:	IV4.M2	0	1.000000E+00
		0	1.000000E+00
calf:	IV4.L09		
mother:	IV4.F1		
male:	IV4.M2	0	1.000000E+00
		0	1.000000E+00
		all	1.000000E+00
calf:	IV4.L10		
mother:	IV4.F1		
male:	IV4.M2	0	1.000000E+00
		0	1.000000E+00
		all	1.000000E+00
calf:	IV4.L11		
mother:	IV4.F1		
male:	IV4.M2	0	1.000000E+00
		0	1.000000E+00
		all	1.000000E+00
calf:	IV4.L12		
mother:	IV4.F1		
male:	IV4.M2	0	1.000000E+00
		0	1.000000E+00
		all	1.000000E+00
calf:	IV4.L13		
mother:	IV4.F1		
male:	IV4.M2	0	1.000000E+00
		0	1.000000E+00
		all	1.000000E+00
calf:	IV4.L14		
mother:	IV4.F1		
male:	IV4.M2	0	1.000000E+00
		0	1.000000E+00
		all	1.000000E+00
calf:	IV4.L15		
mother:	IV4.F1		
male:	IV4.M2	0	1.000000E+00
		0	1.000000E+00
		all	1.000000E+00
calf:	IV4.L16		
mother:	IV4.F1		
male:	IV4.M2	0	1.000000E+00
		0	1.000000E+00
		all	1.000000E+00
calf:	IV4.L27		
mother:	IV4.F1		
male:	IV4.M2	0	1.000000E+00
		0	1.000000E+00

labels:		group:	P(father)
		all	1.000000E+00
calf:	IV4.L18		
mother:	IV4.F1		
male:	IV4.M2	0	1.000000E+00
		0	1.000000E+00
		all	1.000000E+00
calf:	IV4.L19		
mother:	IV4.F1		
male:	IV4.M2	0	1.000000E+00
		0	1.000000E+00
		all	1.000000E+00
calf:	IV4.L20		
mother:	IV4.F1		
male:	IV4.M2	0	1.000000E+00
		0	1.000000E+00
		all	1.000000E+00
calf:	IV4.L21		
mother:	IV4.F1		
male:	IV4.M2	0	1.000000E+00
		0	1.000000E+00
		all	1.000000E+00
calf:	IV4.L22		
mother:	IV4.F1		
male:	IV4.M2	0	1.000000E+00
		0	1.000000E+00
		all	1.000000E+00
calf:	IV4.L23		
mother:	IV4.F1		
male:	IV4.M2	0	1.000000E+00
		0	1.000000E+00
		all	1.000000E+00
calf:	IV4.L24		
mother:	IV4.F1		
male:	IV4.M2	0	1.000000E+00
		0	1.000000E+00
		all	1.000000E+00
calf:	IV4.L25		
mother:	IV4.F1		
male:	IV4.M2	0	1.000000E+00
		0	1.000000E+00
		all	1.000000E+00
calf:	IV4.L26		
mother:	IV4.F1		
male:	IV4.M2	0	1.000000E+00
		0	1.000000E+00
		all	1.000000E+00
calf:	IV4.L37		
mother:	IV4.F1		
male:	IV4.M2	0	1.000000E+00
		0	1.000000E+00
		all	1.000000E+00

labels:		group:	P(father)
		all	1.000000E+00
calf:	IV4.L28		
mother:	IV4.F1		
male:	IV4.M2	0	1.000000E+00
		0	1.000000E+00
		all	1.000000E+00
calf:	IV4.L29		
mother:	IV4.F1		
male:	IV4.M2	0	1.000000E+00
		0	1.000000E+00
		all	1.000000E+00
calf:	IV4.L30		
mother:	IV4.F1		
male:	IV4.M2	0	1.000000E+00
		0	1.000000E+00
		all	1.000000E+00
calf:	IV4.L31		
mother:	IV4.F1		
male:	IV4.M2	0	1.000000E+00
		0	1.000000E+00
		all	1.000000E+00
calf:	IV4.L32		
mother:	IV4.F1		
male:	IV4.M2	0	1.000000E+00
		0	1.000000E+00
		all	1.000000E+00
calf:	IV4.L33		
mother:	IV4.F1		
male:	IV4.M2	0	1.000000E+00
		0	1.000000E+00
		all	1.000000E+00
calf:	IV4.L34		
mother:	IV4.F1		
male:	IV4.M2	0	1.000000E+00
		0	1.000000E+00
		all	1.000000E+00
calf:	IV4.L35		
mother:	IV4.F1		
male:	IV4.M2	0	1.000000E+00
		0	1.000000E+00
		all	1.000000E+00
calf:	IV4.L36		
mother:	IV4.F1		
male:	IV4.M2	0	1.000000E+00
		0	1.000000E+00
		all	1.000000E+00
calf:	IV4.L47		
mother:	IV4.F1		
male:	IV4.M2	0	1.000000E+00
		0	1.000000E+00
		all	1.000000E+00

labels:		group:	P(father)
calf:	IV4.L38		
mother:	IV4.F1		
male:	IV4.M2	0	1.000000E+00
		0	1.000000E+00
		all	1.000000E+00
calf:	IV4.L39		
mother:	IV4.F1		
male:	IV4.M1	0	1.000000E+00
		0	1.000000E+00
		all	1.000000E+00
calf:	IV4.L40		
mother:	IV4.F1		
male:	IV4.M2	0	1.000000E+00
		0	1.000000E+00
		all	1.000000E+00
calf:	IV4.L41		
mother:	IV4.F1		
male:	IV4.M2	0	1.000000E+00
		0	1.000000E+00
		all	1.000000E+00
calf:	IV4.L42		
mother:	IV4.F1		
male:	IV4.M2	0	1.000000E+00
		0	1.000000E+00
		all	1.000000E+00
calf:	IV4.L43		
mother:	IV4.F1		
male:	IV4.M2	0	1.000000E+00
		0	1.000000E+00
		all	1.000000E+00
calf:	IV4.L44		
mother:	IV4.F1		
male:	IV4.M2	0	1.000000E+00
		0	1.000000E+00
		all	1.000000E+00
calf:	IV4.L45		
mother:	IV4.F1		
male:	IV4.M2	0	1.000000E+00
		0	1.000000E+00
		all	1.000000E+00
calf:	IV4.L46		
mother:	IV4.F1		
male:	IV4.M2	0	1.000000E+00
		0	1.000000E+00
		all	1.000000E+00
calf:	IV4.L57		
mother:	IV4.F1		
male:	IV4.M2	0	1.000000E+00
		0	1.000000E+00
		all	1.000000E+00

labels:		group:	P(father)
calf:	IV4.L48		
mother:	IV4.F1		
male:	IV4.M2	0	1.000000E+00
		0	1.000000E+00
		all	1.000000E+00
calf:	IV4.L49		
mother:	IV4.F1		
male:	IV4.M2	0	1.000000E+00
		0	1.000000E+00
		all	1.000000E+00
calf:	IV4.L50		
mother:	IV4.F1		
male:	IV4.M2	0	1.000000E+00
		0	1.000000E+00
		all	1.000000E+00
calf:	IV4.L51		
mother:	IV4.F1		
male:	IV4.M2	0	1.000000E+00
		0	1.000000E+00
		all	1.000000E+00
calf:	IV4.L52		
mother:	IV4.F1		
male:	IV4.M2	0	1.000000E+00
		0	1.000000E+00
		all	1.000000E+00
calf:	IV4.L53		
mother:	IV4.F1		
male:	IV4.M2	0	1.000000E+00
		0	1.000000E+00
		all	1.000000E+00
calf:	IV4.L54		
mother:	IV4.F1		
male:	IV4.M2	0	1.000000E+00
		0	1.000000E+00
		all	1.000000E+00
calf:	IV4.L55		
mother:	IV4.F1		
male:	IV4.M2	0	1.000000E+00
		0	1.000000E+00
		all	1.000000E+00
calf:	IV4.L56		
mother:	IV4.F1		
male:	IV4.M2	0	1.000000E+00
		0	1.000000E+00
		all	1.000000E+00
calf:	IV4.L67		
mother:	IV4.F1		
male:	IV4.M2	0	1.000000E+00
		0	1.000000E+00
		all	1.000000E+00

labels:		group:	P(father)
calf:	IV4.L58		
mother:	IV4.F1		
male:	IV4.M2	0	1.000000E+00
		0	1.000000E+00
		all	1.000000E+00
calf:	IV4.L59		
mother:	IV4.F1		
male:	IV4.M2	0	1.000000E+00
		0	1.000000E+00
		all	1.000000E+00
calf:	IV4.L60		
mother:	IV4.F1		
male:	IV4.M2	0	1.000000E+00
		0	1.000000E+00
		all	1.000000E+00
calf:	IV4.L61		
mother:	IV4.F1		
male:	IV4.M2	0	1.000000E+00
		0	1.000000E+00
		all	1.000000E+00
calf:	IV4.L62		
mother:	IV4.F1		
male:	IV4.M2	0	1.000000E+00
		0	1.000000E+00
		all	1.000000E+00
calf:	IV4.L63		
mother:	IV4.F1		
male:	IV4.M2	0	1.000000E+00
		0	1.000000E+00
		all	1.000000E+00
calf:	IV4.L64		
mother:	IV4.F1		
male:	IV4.M2	0	1.000000E+00
		0	1.000000E+00
		all	1.000000E+00
calf:	IV4.L65		
mother:	IV4.F1		
male:	IV4.M2	0	1.000000E+00
		0	1.000000E+00
		all	1.000000E+00
calf:	IV4.L66		
mother:	IV4.F1		
male:	IV4.M2	0	1.000000E+00
		0	1.000000E+00
		all	1.000000E+00
calf:	IV4.L77		
mother:	IV4.F1		
male:	IV4.M2	0	1.000000E+00
		0	1.000000E+00
		all	1.000000E+00
calf:	IV4.L78		

labels:		group:	P(father)
calf:	IV4.L68		
mother:	IV4.F1		
male:	IV4.M2	0	1.000000E+00
		0	1.000000E+00
		all	1.000000E+00
calf:	IV4.L69		
mother:	IV4.F1		
male:	IV4.M2	0	1.000000E+00
		0	1.000000E+00
		all	1.000000E+00
calf:	IV4.L70		
mother:	IV4.F1		
male:	IV4.M2	0	1.000000E+00
		0	1.000000E+00
		all	1.000000E+00
calf:	IV4.L71		
mother:	IV4.F1		
male:	IV4.M2	0	1.000000E+00
		0	1.000000E+00
		all	1.000000E+00
calf:	IV4.L72		
mother:	IV4.F1		
male:	IV4.M2	0	1.000000E+00
		0	1.000000E+00
		all	1.000000E+00
calf:	IV4.L73		
mother:	IV4.F1		
male:	IV4.M2	0	1.000000E+00
		0	1.000000E+00
		all	1.000000E+00
calf:	IV4.L74		
mother:	IV4.F1		
male:	IV4.M2	0	1.000000E+00
		0	1.000000E+00
		all	1.000000E+00
calf:	IV4.L75		
mother:	IV4.F1		
male:	IV4.M2	0	1.000000E+00
		0	1.000000E+00
		all	1.000000E+00
calf:	IV4.L76		
mother:	IV4.F1		
male:	IV4.M2	0	1.000000E+00
		0	1.000000E+00
		all	1.000000E+00
calf:	IV4.L87		
mother:	IV4.F1		
male:	IV4.M2	0	1.000000E+00
		0	1.000000E+00
		all	1.000000E+00
calf:	IV4.L88		

**labels:**		**group:**	**P(father)**
mother:	IV4.F1		
male:	IV4.M2	0	1.000000E+00
		0	1.000000E+00
		all	1.000000E+00
calf:	IV4.L79		
mother:	IV4.F1		
male:	IV4.M2	0	1.000000E+00
		0	1.000000E+00
		all	1.000000E+00
calf:	IV4.L80		
mother:	IV4.F1		
male:	IV4.M2	0	1.000000E+00
		0	1.000000E+00
		all	1.000000E+00
calf:	IV4.L81		
mother:	IV4.F1		
male:	IV4.M2	0	1.000000E+00
		0	1.000000E+00
		all	1.000000E+00
calf:	IV4.L82		
mother:	IV4.F1		
male:	IV4.M2	0	1.000000E+00
		0	1.000000E+00
		all	1.000000E+00
calf:	IV4.L83		
mother:	IV4.F1		
male:	IV4.M2	0	1.000000E+00
		0	1.000000E+00
		all	1.000000E+00
calf:	IV4.L84		
mother:	IV4.F1		
male:	IV4.M2	0	1.000000E+00
		0	1.000000E+00
		all	1.000000E+00
calf:	IV4.L85		
mother:	IV4.F1		
male:	IV4.M1	0	1.000000E+00
		0	1.000000E+00
		all	1.000000E+00
calf:	IV4.L86		
mother:	IV4.F1		
male:	IV4.M2	0	1.000000E+00
		0	1.000000E+00
		all	1.000000E+00
calf:	IV4.L97		
mother:	IV4.F1		
male:	IV4.M2	0	1.000000E+00
		0	1.000000E+00
		all	1.000000E+00
calf:	IV4.L98		
mother:	IV4.F1		

**labels:**		**group:**	**P(father)**
mother:	IV4.F1		
male:	IV4.M2	0	1.000000E+00
		0	1.000000E+00
		all	1.000000E+00
calf:	IV4.L89		
mother:	IV4.F1		
male:	IV4.M2	0	1.000000E+00
		0	1.000000E+00
		all	1.000000E+00
calf:	IV4.L90		
mother:	IV4.F1		
male:	IV4.M2	0	1.000000E+00
		0	1.000000E+00
		all	1.000000E+00
calf:	IV4.L91		
mother:	IV4.F1		
male:	IV4.M2	0	1.000000E+00
		0	1.000000E+00
		all	1.000000E+00
calf:	IV4.L92		
mother:	IV4.F1		
male:	IV4.M2	0	1.000000E+00
		0	1.000000E+00
		all	1.000000E+00
calf:	IV4.L93		
mother:	IV4.F1		
male:	IV4.M2	0	1.000000E+00
		0	1.000000E+00
		all	1.000000E+00
calf:	IV4.L94		
mother:	IV4.F1		
male:	IV4.M2	0	1.000000E+00
		0	1.000000E+00
		all	1.000000E+00
calf:	IV4.L95		
mother:	IV4.F1		
male:	IV4.M1	0	1.000000E+00
		0	1.000000E+00
		all	1.000000E+00
calf:	IV4.L96		
mother:	IV4.F1		
male:	IV4.M2	0	1.000000E+00
		0	1.000000E+00
		all	1.000000E+00
calf:	IV4.L107		
mother:	IV4.F1		
male:	IV4.M2	0	1.000000E+00
		0	1.000000E+00
		all	1.000000E+00
calf:	IV4.L108		
mother:	IV4.F1		

labels:		group:	P(father)
male:	IV4.M2	0	1.000000E+00
		0	1.000000E+00
		all	1.000000E+00
calf:	IV4.L99		
mother:	IV4.F1		
male:	IV4.M2	0	1.000000E+00
		0	1.000000E+00
		all	1.000000E+00
calf:	IV4.L100		
mother:	IV4.F1		
male:	IV4.M2	0	1.000000E+00
		0	1.000000E+00
		all	1.000000E+00
calf:	IV4.L101		
mother:	IV4.F1		
male:	IV4.M2	0	1.000000E+00
		0	1.000000E+00
		all	1.000000E+00
calf:	IV4.L102		
mother:	IV4.F1		
male:	IV4.M2	0	1.000000E+00
		0	1.000000E+00
		all	1.000000E+00
calf:	IV4.L103		
mother:	IV4.F1		
male:	IV4.M2	0	1.000000E+00
		0	1.000000E+00
		all	1.000000E+00
calf:	IV4.L104		
mother:	IV4.F1		
male:	IV4.M2	0	1.000000E+00
		0	1.000000E+00
		all	1.000000E+00
calf:	IV4.L105		
mother:	IV4.F1		
male:	IV4.M1	0	1.000000E+00
0	1.000000E+00		
all	1.000000E+00		
calf:	IV4.L106		
mother:	IV4.F1		
male:	IV4.M2	0	1.000000E+00
		0	1.000000E+00
		all	1.000000E+00
calf:	IV4.L117		
mother:	IV4.F1		
male:	IV4.M2	0	1.000000E+00
		0	1.000000E+00
		all	1.000000E+00
calf:	IV4.L118		
mother:	IV4.F1		
male:	IV4.M2	0	1.000000E+00

labels:		group:	P(father)
male:	IV4.M2	0	1.000000E+00
		0	1.000000E+00
		all	1.000000E+00
calf:	IV4.L109		
mother:	IV4.F1		
male:	IV4.M2	0	1.000000E+00
		0	1.000000E+00
		all	1.000000E+00
calf:	IV4.L110		
mother:	IV4.F1		
male:	IV4.M2	0	1.000000E+00
		0	1.000000E+00
		all	1.000000E+00
calf:	IV4.L111		
mother:	IV4.F1		
male:	IV4.M2	0	1.000000E+00
		0	1.000000E+00
		all	1.000000E+00
calf:	IV4.L112		
mother:	IV4.F1		
male:	IV4.M2	0	1.000000E+00
		0	1.000000E+00
		all	1.000000E+00
calf:	IV4.L113		
mother:	IV4.F1		
male:	IV4.M2	0	1.000000E+00
		0	1.000000E+00
		all	1.000000E+00
calf:	IV4.L114		
mother:	IV4.F1		
male:	IV4.M2	0	1.000000E+00
		0	1.000000E+00
		all	1.000000E+00
calf:	IV4.L115		
mother:	IV4.F1		
male:	IV4.M1	0	1.000000E+00
		0	1.000000E+00
		all	1.000000E+00
calf:	IV4.L116		
mother:	IV4.F1		
male:	IV4.M2	0	1.000000E+00
		0	1.000000E+00
		all	1.000000E+00

labels:		group:	P(father)	labels:	group:	P(father)
		0	1.000000E+00			
		all	1.000000E+00			
calf:	IV4.L119					
mother:	IV4.F1					
male:	IV4.M2	0	1.000000E+00			
		0	1.000000E+00			
		all	1.000000E+00			
calf:	IV4.L120					
mother:	IV4.F1					
male:	IV4.M2	0	1.000000E+00			
		0	1.000000E+00			
		all	1.000000E+00			
calf:	IV4.L121					
mother:	IV4.F1					
male:	IV4.M2	0	1.000000E+00			
		0	1.000000E+00			
		all	1.000000E+00			
calf:	IV4.L122					
mother:	IV4.F1					
male:	IV4.M2	0	1.000000E+00			
		0	1.000000E+00			
		all	1.000000E+00			
calf:	IV4.L123					
mother:	IV4.F1					
male:	IV4.M2	0	1.000000E+00			
		0	1.000000E+00			
		all	1.000000E+00			
calf:	IV4.L124					
mother:	IV4.F1					
male:	IV4.M2	0	1.000000E+00			
		0	1.000000E+00			
		all	1.000000E+00			
calf:	IV4.L125					
mother:	IV4.F1					
male:	IV4.M1	0	1.000000E+00			
		0	1.000000E+00			
		all	1.000000E+00			

Legend:

<offspring ID>
<mother ID>
<male x ID> <group of male x> <Probability of male x is the father>
<group y> < Probability of male x belongs to group y>
all < Probability of male x has been sampled>
